# Self-assembled albumin nanoparticles induce pyroptosis for photodynamic/photothermal/immuno synergistic therapies in triple-negative breast cancer

**DOI:** 10.3389/fimmu.2023.1173487

**Published:** 2023-05-26

**Authors:** Jianquan Yang, Wen Guo, Rong Huang, Jiaojiao Bian, Siqi Zhang, Ting Wei, Chuanshi He, Ziyue Hu, Juan Li, Chunyang Zhou, Man Lu

**Affiliations:** ^1^ School of Medicine, University of Electronic Science and Technology of China, Chengdu, Sichuan, China; ^2^ Department of Ultrasound Medical Center, Sichuan Cancer Hospital & Institute, Sichuan Cancer Center, School of Medicine, University of Electronic Science and Technology of China, Chengdu, Sichuan, China; ^3^ Institute of Materia Medica, North Sichuan Medical College, Nanchong, Sichuan, China

**Keywords:** triple-negative breast cancer, pyroptosis, phototherapy, immunotherapy, nanomedicine

## Abstract

Triple-negative breast cancer (TNBC) is characterized by a high degree of malignancy, early metastasis, limited treatment, and poor prognosis. Immunotherapy, as a new and most promising treatment for cancer, has limited efficacy in TNBC because of the immunosuppressive tumor microenvironment (TME). Inducing pyroptosis and activating the cyclic guanosine monophosphate-adenosine monophosphate synthase/interferon gene stimulator (cGAS/STING) signaling pathway to upregulate innate immunity have become an emerging strategy for enhancing tumor immunotherapy. In this study, albumin nanospheres were constructed with photosensitizer-IR780 encapsulated in the core and cGAS–STING agonists/H_2_S producer-ZnS loaded on the shell (named IR780-ZnS@HSA). *In vitro*, IR780-ZnS@HSA produced photothermal therapy (PTT) and photodynamic therapy (PDT) effects. In addition, it stimulated immunogenic cell death (ICD) and activated pyroptosis in tumor cells *via* the caspase-3–GSDME signaling pathway. IR780-ZnS@HSA also activated the cGAS–STING signaling pathway. The two pathways synergistically boost immune response. *In vivo*, IR780-ZnS@HSA + laser significantly inhibited tumor growth in 4T1 tumor-bearing mice and triggered an immune response, improving the efficacy of the anti-APD-L1 antibody (aPD-L1). In conclusion, IR780-ZnS@HSA, as a novel inducer of pyroptosis, can significantly inhibit tumor growth and improve the efficacy of aPD-L1.

## Introduction

Breast cancer has the highest incidence and mortality in women (1). Triple-negative breast cancer (TNBC), a type of breast cancer where estrogen receptor (ER), progesterone receptor (PR), and Her-2 are expressed at a low level, has the highest malignancy and worst breast cancer prognoses (2). Due to the lack of specific therapeutic targets, the current treatment for TNBC primarily includes surgery, chemotherapy, and radiotherapy (3). Therefore, it is urgent to find new treatments to improve TNBC prognosis (4).

As an emerging method in treating malignant tumors, immunotherapy has achieved specific therapeutic effects in melanoma, lung cancer, and other tumors (5–7). However, immunotherapy has a limited effect on breast cancer, especially in TNBC. Previous studies have shown that tumor microenvironment (TME) plays an essential role in immunotherapy. The efficacy of immunotherapy is limited in TNBC because immune cells, especially CD8^+^ T cells, cannot effectively infiltrate tumor tissues due to the immunosuppressive microenvironment. Hence, antitumor immunity has become a research hotspot in TNBC (8, 9).

Phototherapy is a promising strategy for cancer treatment by reactive oxygen species (ROS) in photodynamic therapy (PDT) and by hyperthermia in photothermal therapy (PTT) (5). Due to its non-invasive and specific characteristics, PTT/PDT is considered a promising cancer treatment (6, 7). Indeed, a series of studies have shown that PTT/PDT can induce immunogenic cell death (ICD) in tumor cells, release tumor antigens, promote the infiltration of immune cells to the tumor site, and enhance the efficacy of immunotherapy (8, 9).

Pyroptosis is a newly discovered inflammation-programmed cell death that differs from traditional non-inflammatory cell death, such as apoptosis (10, 11). Numerous studies have shown the relationship between pyroptosis and tumor immunotherapy. Pyroptosis can boost the immune response and enhance the effect of immunotherapy by releasing various inflammatory cytokines, such as IL-1β (12, 13). It has been shown that double-strand DNA (dsDNA) increases in the cytoplasm and binds to cyclic GMP-AMP synthase (cGAS) during tumorigenesis. This is followed by interferon gene (STING) activation, which induces the expression of type I interferon (IFN) and other proinflammatory cytokines, leading to dendritic cell (DC) recruitment, cytotoxic T cell infiltration, and innate immunity activation (14–17). Because of the versatility and exclusive properties of nanomaterials, therapeutic techniques based on nanoparticles are being developed and have attracted attention (18–20). Pyroptosis and cGAS–STING pathways play an essential role in enhancing tumor immunotherapy. Multifunctional nanoparticles are designed to play a role in tumor immunotherapy by inducing pyroptosis and activating the cGAS–STING pathways.

**Figure 1:**
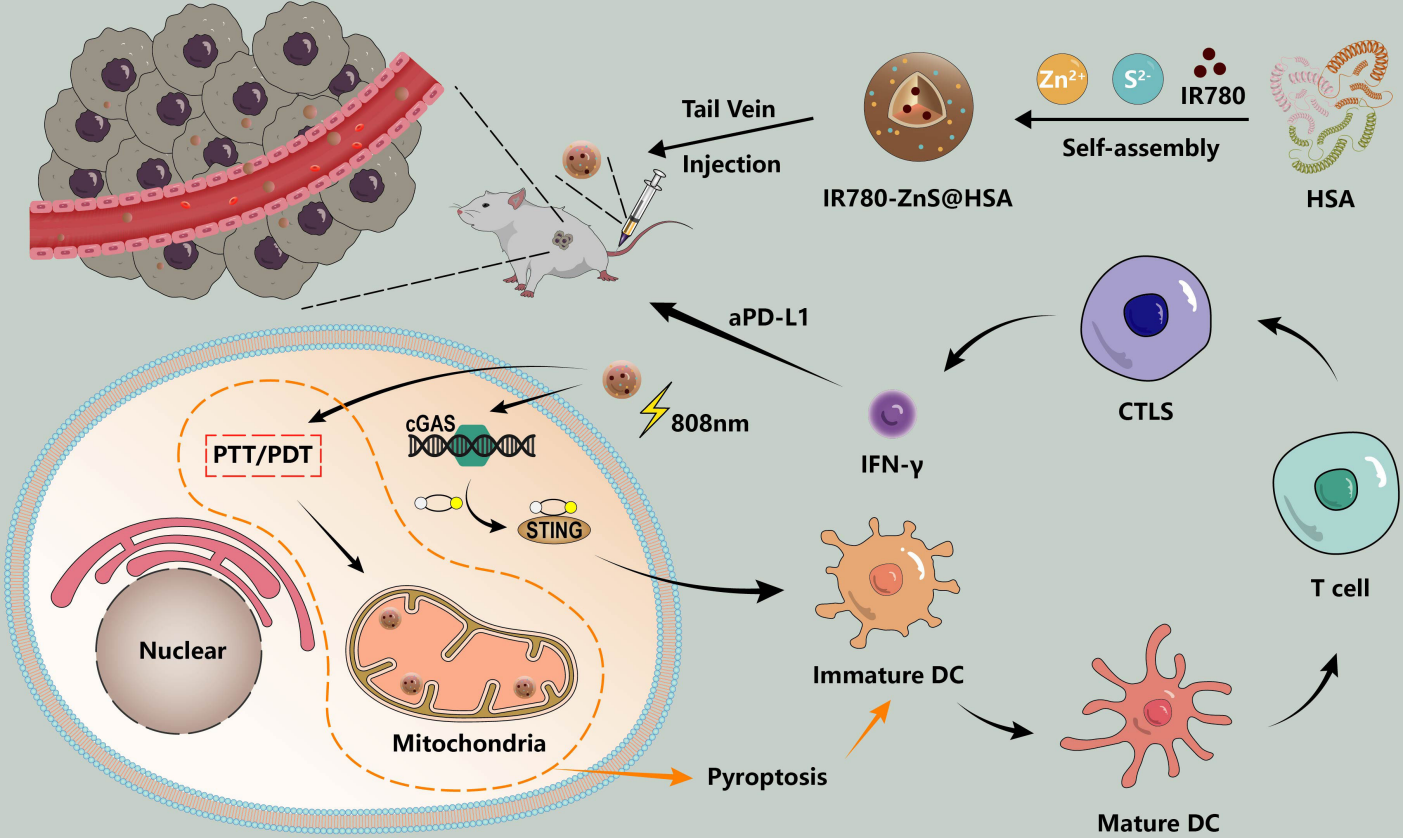
Schematic illustration of the synthesis and therapeutic process of IR780-ZnS@HSA nanoparticles (NPs). cGAS–STING agonist -ZnS and PS-IR780 were co-loaded into HSA (named IR780-ZnS@HSA) to achieve local PT and systemic immune response. Local PT and mitigation of immunosuppression (PD-1/PD-L1 checkpoint blocking and cGAS–STING pathway activation) are highly effective strategies for triple-negative breast cancer (TNBC) treatment.

In this study, target-specific HSA was used as the nanocarrier (21), holding IR780, the mitochondria-specific targeting PTT/PDT drugs (22, 23), and ZnS, the cGAS–STING agonist. The H2S generator (24) was synthesized by a self-assembly approach to generate IR780-ZnS@HSA, which has a trinity function of PTT/PDT/immunotherapy. In vitro, IR780-ZnS@HSA activated the cGAS–STING pathway and induced ICD and pyroptosis in tumor cells. In vivo, IR780-ZnS@HSA significantly inhibited tumor growth in 4T1 tumor-bearing balb/c mice. Interestingly, IR780-ZnS@HSA changed the immunosuppressive TME and boosted the immune response. IR780-ZnS@HSA combined with aPD-L1 can inhibit the growth of primary and distant metastatic tumors in 4T1 tumor-bearing mice (**Figure 1**).

## Materials and methods

### Materials

IR780, HSA, zinc acetate, and sodium sulfide were purchased from Solarbio (Beijing, China). Fetal bovine serum (FBS) and Dulbecco’s modified Eagle medium (DMEM) were purchased from GIBCO (Grand Island, NY, USA). Mito-Tracker Green was purchased from Beyotime (Shanghai, China; #C1048). Annexin V-FITC Apoptosis Kit (BD, San Jose, CA, USA; #556557) was from Becton, Dickinson and Company (Franklin Lakes, NJ, USA). Anti-DFNA5/GSDME-N-terminal (Abcam, Cambridge, UK; #AB215191), anti-calreticulin antibody (Abcam, #ab92516), anti-high-mobility group box 1 (HMGB1) antibody (Abcam, #ab79823), anti-calreticulin antibody (Abcam, ab92516), goat anti-rabbit IgG H&L (Alexa Fluor 488) (Abcam, #ab150081), anti-CD8 antibody (Abcam, #ab217344), anti-FOXP3 (Abcam, #ab20034) were from Abcam. Cleaved caspase-3 (CST, Danvers, MA, USA; #9664T), cGAS (CST, #31659S), STING (CST, #50494S), and p-STING (CST, #50907) were from Cell Signaling Technology (Danvers, MA, USA). Atezolizumab (Selleck, Munich, Germany; #A2004) were from Selleck. Mouse IFN-β ELISA Kit (Beyotime, #PI568) and human IFN-β ELISA Kit (Beyotime, #PI572) were from Beyotime. SOSG9 (Meilunbio, Dalian, China; #MA0326) was from Meilunbio.

### Synthesis of nanoparticles

The synthesis of IR780-ZnS@HSA is slightly modified based on previous study reports (21, 24, 25); that is, 40.0 mg of HSA was dissolved in 6 ml of deionized water, then triethylamine 2 μl was added by continuous stirring, followed by the addition of 1.0 ml of IR780 (2.0 mg/ml), which was dissolved in methanol and then continuously stirred overnight in the dark. The next morning, 1 ml of Zn(CH_3_COO)_2_ solution (13.75 mg/ml) was added to the liquid by drops, followed by 1 ml of Na_2_S solution (18.2 mg/ml). The mixture was stirred at a high speed for 4 h and then filtered through an ultrafiltration tube (MW: 8,000–14,000) to obtain IR780-ZnS@HSA. IR780@HSA and ZnS@HSA were also performed according to the above method, except that Zn(CH_3_COO)_2_, Na_2_S, and IR780 were not added.

### Characteristics of nanoparticles

The diameter and zeta potential of the nanoparticles (NPs) were measured with Malvern Zetasizer Nano ZS90 (Worcestershire, UK). The morphology of NPs was observed by transmission electron microscopy (TEM).

### Stability of IR780-ZnS@HSA and drug release

To evaluate the stability of the NPs, 500 μl of NP solution was mixed with 500 μl of FBS. The size and zeta potential of the mixture were measured after incubation at room temperature for 2, 6, 12, 24, and 48 h. The release degree of IR780-ZnS@HSA NPs under different pH and temperature conditions was detected by the dialysis method (26).

### Photothermal effect In vitro

To investigate the photothermal effects of NPs, 1 ml of NP solution (IR780-ZnS@HSA, IR780@HSA, and ZnS@HSA) and phosphate-buffered saline (PBS) was irradiated with PBS exposure to 808 nm laser irradiation at the power of 1 W/cm^2^ for 10 min. To evaluate the Light stability, the temperature curve of the nanoparticle solution was measured after four cycles of 808 nm laser on/off. The temperature change of the solution was monitored by a thermal infrared camera and quantified by the FLIR Tools.

### Detection of singlet oxygen generation

The IR780-ZnS@HSA solution (IR780 concentration 8 μg/ml) and SOSG fluorescent probe were added to the 6-well plate. Then, the solution was thoroughly mixed and exposed to 808 nm laser (1 W/cm^2^) for irradiation for 5 min. After irradiation, the solution was collected in the cuvette immediately, and the fluorescence emission spectrum of SOSG was detected by a fluorescence spectrophotometer (excitation wavelength = 504 nm, emission wavelength = 525 nm) (27).

### Cell lines and animals

Human TNBC cell line MDA-MB-231, mouse TNBC cell line 4T1, and human umbilical vein endothelial cell (HUVEC) were purchased from ATCC (Manassas, VA, USA) and cultured in DMEM supplemented with 10% FBS (GIBCO) and 100 U/ml of penicillin–streptomycin. Cells were cultured in a 37°C incubator in a 5% CO_2_ atmosphere. Female balb/c mice (6–8 weeks old) were purchased from Beijing Huafukang Bioscience Co. Inc. (Beijing, China) and maintained in the animal house of Sichuan Cancer Hospital. The animal study was reviewed and approved by Sichuan Cancer Hospital & Institute, Sichuan Cancer Center, Affiliated Cancer Hospital of the University of Electronic Science and Technology of China (SCCHEC-04-2020-004).

### NP uptake by cancer cells

MDA-MB-231 cells and 4T1 cells were seeded into 12-well plates with 5 × 10^4^ cells per well. After cell adhesion, NPs were added, and then the NP uptake was observed by confocal laser scanning microscopy (CLSM) and flow cytometry at 2 and 4 h, respectively.

### Mitochondrial targeting and co-localization of the mitochondria

MDA-MB-231 cells and 4T1 cells were seeded in 12-well plates with 5 × 10^4^ cells per well and co-culture with different NPs. Mito-Tracker Green is used according to the manufacturer’s instructions (Beyotime, China; C1048). The targeting of mitochondria of NPs was observed by CLSM.

### Intracellular ROS assay

MDA-MB-231 cells and 4T1 cells were inoculated in 12-well plates with 5 × 10^4^ cells and treated with different NPs. The Reactive Oxygen Species Detection Kit (DCFH-DA) was used according to the manufacturer’s instructions (Beyotime, China; S0033S). CLSM was used to observe the ROS production in tumor cells.

### Cell proliferation assay

MDA-MB-231 cells and 4T1 cells were cultured in 96-well with 5 × 10^3^ cells per well. Different NPs were added and co-cultured with tumor cells at 37°C for 24 h. The cells were irradiated by 808 nm laser irradiation with the power of 1 W/cm^2^ for 5 min. After 24 h, Cell Counting Kit-8 solution (BS350B; Biosharp, Hefei, Anhui, China) was diluted with medium and incubated at a ratio of 1:10 for 2 h. The optical density was measured by the absorbance of the wavelength at 450 nm.

### Mitochondrial membrane potential assay

MDA-MB-231 cells and 4T1 cells were treated with different nanoparticles for 24 h. The cells were washed twice with PBS and incubated in the dark at 37°C for 30 min according to the method described in the reagent manufacturer’s instructions (JC-1, Beyotime, China; C2003S). Flow cytometry (BD, FACSCanto II) was used to detect the red and green fluorescence intensity of cells.

### Apoptosis assay

Annexin V-FITC Apoptosis Kit (BD, #556557, USA) was used to detect apoptosis in tumor cells. The test for apoptosis was according to the manufacturer’s instructions. MDA-MB-231 cells and 4T1 cells were treated with different nanoparticles for 24 h, washed twice with cold PBS, and then incubated with PE and Annexin V-FITC at room temperature for 15 min. Cell apoptosis was detected by flow cytometry (BD, FACSCanto II).

### Western blotting

MDA-MB-231 cells and 4T1 cells were treated with different NPs with or without irradiation by 808 nm laser with the power of 1 W/cm^2^ for 5 min. After 24 h, tumor cells were collected and lysed using a radioimmunoprecipitation assay buffer and phenylmethyl sulfonyl fluoride mixture (100:1 ratio) on ice for 30 min at 15,000 RPM core separation for 30 min. The supernatant was added and heated at 100°C for 10 min. It was separated by sodium dodecyl sulfate–polyacrylamide gel electrophoresis (SDS-PAGE) and transferred to a polyvinylidene chloride difluoride membrane. The membranes were observed by enhanced chemiluminescence. The main antibodies were GSDMD, GSDME, GSDMD-N, GSMDE-N, caspase-1, cleaved caspase-1, cleaved caspase-3, STING, cGAS, and p-STING.

### ELISA experiment

MDA-MB-231 and 4T1 cells were treated with different NPs with or without irradiation by 808 nm laser with the power of 1 W/cm^2^ for 5 min. After 24 h, the supernatant of tumor cells in different groups was collected to detect the IL-18 (FineTest, Wuhan, China; #EH0011 and EM1185), IL-1β (FineTest, China; #EM0109 and EH0185), and IFN-β (Beyotime, China; #PI568 and #PI572) by ELISA kit according to the manufacturer’s instruction. In vivo, serum cytokine levels of IFN-γ (Zcibio, Shanghai, China; #ZC-37905) and TNF-α (Zcibio, China; #ZC-39024) were performed on day 3 after various treatments in by ELISA kit according to the manufacturer’s instruction.

### RNA sequencing

4T1 cells and MDA-MB-231 cells were planted in six-well plates with a density of 5 × 10^5^/well. In the experimental group, IR780-ZnS@HSA was co-incubated for 4 h and followed by 808 nm laser irradiation with the power of 1 W/cm^2^ for 5 min. In the control group, only PBS replaced IR780-ZnS@HSA, and other treatments were consistent with the experimental group. After 24 h of irradiation, cells were repeatedly cleaned with PBS and collected for RNA sequencing (Novogene, Beijing, China).

### Hemolysis test

The hemolysis experiment was slightly modified according to the previous experimental scheme (27). The red blood cell (RBC) suspension was incubated with different concentrations of IR780-ZnS@HSA, deionized water (positive control), and PBS (negative control) in the incubator at 37°C for 3 h. The suspension was then centrifuged at 3,000 *g* for 10 min and photographed. The absorbance of the supernatant was measured at 540 nm using a multi-board reader (1510, Thermo Scientific, Waltham, MA, USA). The hemolysis rate can be calculated using the following formula: hemolysis (%) = [(OD sample − OD negative)/(OD positive − OD negative)] × 100% (27).

### In situ antitumor activity

The subcutaneous tumor model of 6-week-old female balb/c mice was established by subcutaneous implantation of 5 × 10^6^ 4T1 cells. When the tumor volume reached approximately 70–100 mm^3^, tumor-bearing mice were randomly allocated into eight groups including i) control group, ii) ZnS@HSA group, iii) IR780@HSA group, iv) IR780-ZnS@HSA group, v) laser group, vi) ZnS@HSA + laser group, vii) IR780@HSA + laser group, and viii) IR780-ZnS@HSA + laser group. NPs were injected every other day for three times, and the laser group was irradiated by 808 nm laser with the power of 1 W/cm^2^ for 10 min. Body mass and tumor volume in each group were recorded every 3 days. On day 25, blood samples were collected from arteria curtails for analysis of complete blood analysis and blood biochemistry. Subsequently, mice were sacrificed, and tumor tissues were collected and preserved in 4% paraformaldehyde for further immunohistochemistry (IHC), immunofluorescence, hematoxylin and eosin (H&E) staining, and TdT-mediated dUTP-nick end labeling (TUNEL) analysis.

To monitor the survival of tumor-bearing mice, the animals were categorized into the abovementioned treatment groups (n = 8 per group). The survival rate was observed for 60 days since tumor inoculation.

### In vivo abscopal effect

The 6-week-old female balb/c mice were subcutaneously implanted into the left dorsal with 5 × 10^6^ 4T1 cells to establish a primary tumor model. Six days after inoculation, 5 × 10^6^ 4T1 cells were implanted subcutaneously into the right dorsal to establish a distant tumor model. When the primary tumor grew to 70–100 mm^3^, the mice were divided into four groups (seven mice in each group): i) control group, ii) IR780-ZnS@HSA + laser group, iii) aPD-L1 group, and iv) IR780-ZnS@HSA + laser + aPD-L1 group. On the ninth day, IR780-ZnS@HSA or PBS was injected through the tail vein, once every other day for three times. Twenty-four hours after the injection, tumor-bearing mice were irradiated 808 nm laser with the power of 1 W/cm^2^ for 5 min. Subsequently, aPD-L1 (atezolizumab, 5 mg/kg, Selleck, #A2004) was administered intravenously 24 h after laser irradiation and once every other day for three times. Body weight and tumor volume in each group were recorded every 3 days. The mice were sacrificed on day 25, and tumor tissues were collected and preserved in 4% paraformaldehyde for further IHC, immunofluorescence, H&E staining, and TUNEL.

### Photothermal effect In vivo

The tumor-bearing mice were established. NPs were injected through the tail vein. Twenty-four hours after injection, the mice were irradiated with 808 nm laser with the power of 1 W/cm^2^ for 10 min. Temperature changes on the tumor site were observed with thermal imagers.

### H&E staining

The paraffin sections were stained with H&E for morphological analysis. According to the manufacturer’s instruction (Beyotime, C0105S, China), paraffin sections were dewaxed with xylene and alcohol of different concentrations. After being stained with hematoxylin staining solution for 3 min, differentiation was performed with 0.8% hydrochloric acid. The sections were stained with eosin for 20 s, rinsed with water for 5 min, and sealed transparently (28).

### Immunofluorescence

Immunofluorescence staining was performed on the different groups of frozen sections. The frozen sections were fixed with 100% methanol for 5 min, incubated with 0.1% Triton X-100 for 5 min, then sealed with 1% bovine serum albumin (BSA) for 1 h, then incubated with different concentrations of primary antibody (HMGB1, calreticulin (CRT), CD8, and FoxP3) overnight according to the manufacturer’s instructions, followed by secondary antibody and staining with DAPI, and observed by CLSM.

### Statistical analysis

GraphPad Prism 7.0 (La Jolla, CA, USA) was used. Statistical significance was determined by one-way analysis of variance or unpaired Student’s *t*-test. All the data used in this study fit the hypothesis requiring statistical testing. All quantitative data are expressed as mean ± standard deviation. p < 0.05 was considered statistically significant.

## Results and discussion

**Figure 2:**
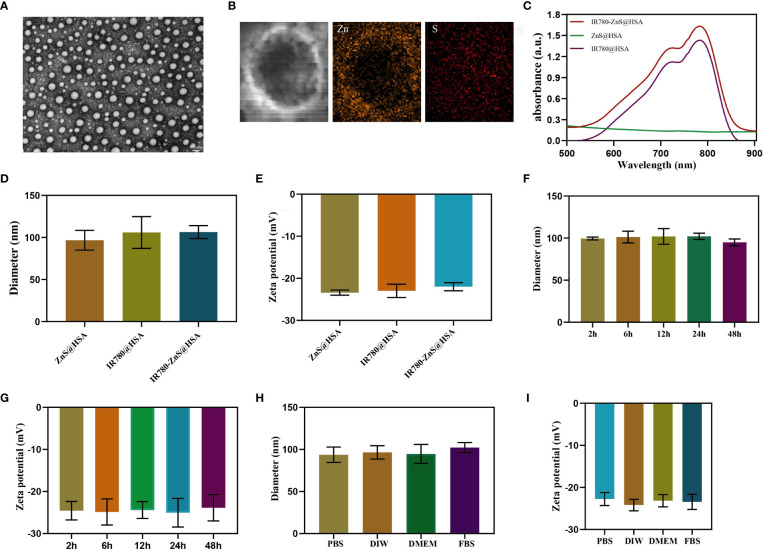
**(A)** Transmission electron microscope (TEM) images of IR780-ZnS@HSA nanoparticles (NPs) (scale bar, 100nm). **(B)** Elemental mapping TEM pictures of IR780-ZnS@HSA NPs. **(C)** UV–vis absorbance spectra and digital photos (inset) of NPs. **(D)** The diameters and **(E)** zeta potentials of NPs. **(F)** The diameters and **(G)** zeta potentials of IR780-ZnS@HSA NPs at different time points. **(H)** The diameters and **(I)** zeta potentials of IR780-ZnS@HSA NPs in various solvents, including phosphate-buffered saline (PBS), deionized water (DIW), Dulbecco’s modified Eagle medium (DMEM), and fetal bovine serum (FBS).

**Figure 3:**
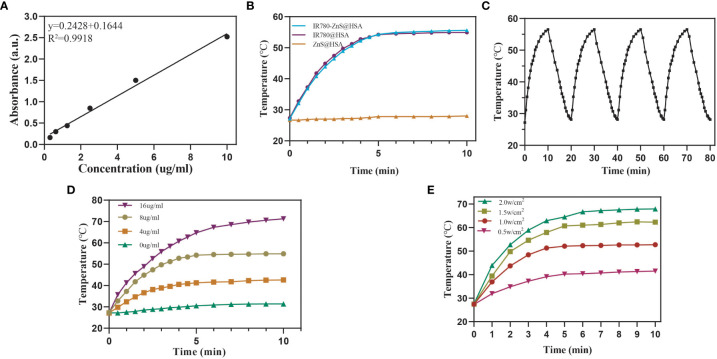
**(A)** Standard curve based on the ultraviolet absorption spectrum of IR780. **(B)** Photothermal-heating curves of IR780-ZnS@HSA, IR780@HSA, and ZnS@HSA exposed to 808 nm laser irradiation at the power of 1 W/cm^2^. **(C)** Four heating and cooling cycles of IR780-ZnS@HSA under on-off light irradiation at the power of 1.0 W/cm^2^. **(D)** Photothermal-heating curves of different IR780-ZnS@HSA concentrations under laser irradiation (808 nm, 1 W/cm^2^). **(E)** Photothermal-heating curves of IR780-ZnS@HSA exposed to 808 nm laser irradiation at varied power densities.

Most studies have used the addition of toxic organic solvents in the synthesis of NPs, which may produce adverse effects on the body. The preparation process of NPs is complicated, its preparation is expensive, and its production rate is low, limiting the clinical conversion and application of NPs (29–31). In this study, HSA, a tumor-targeting reagent (24) approved by the Food and Drug Administration (FDA), was used as the nanocarrier of NPs, ensuring its biosafety. Compared with the enhanced permeability and retention (EPR) effect of NPs, IR780-ZnS@HSA has better tumor-targeting effects (32, 33). In this study, the mitochondria-specific photosensitizer IR780 and the cGAS–STING agonist-ZnS were loaded into the HSA vector to form IR780-ZnS@HSA. IR780-ZnS@HSA was verified for successful preparation by self-assembly approach (**Figure 1**). The TEM indicated that IR780-ZnS@HSA displayed a uniform and spherical morphology, with approximately 100-nm diameter (**Figures 2A–D**). Next, the elemental mapping showed that the IR780-ZnS@HSA contained sodium sulfide and S elements (**Figure 2B**), indicating that ZnS was successfully loaded. At the same time, the UV–visible absorption spectroscopy showed that the peak absorbance of IR780-ZnS@HSA was at 780 nm (**Figure 2C**), indicating that IR780 was successfully loaded into HSA, overcoming the drawback of hydrophobicity. The zeta potential was −26.37 ± 2.63 mV (**Figure 2E**). Studies have shown that NPs have good stability in solution when their zeta potential is approximately −30 mV (34). To further test the stability of IR780-ZnS@HSA, the size and zeta potential of IR780-ZnS@HSA were measured after 2, 6, 12, 24, and 48 h. We observed that the diameter and zeta potential did not change significantly (**Figures 2F,G**). Furthermore, the size and zeta potential of IR780-ZnS@HSA did not change when dissolved in PBS, deionized water (DIW), DMEM, and FBS (**Figures 2H, I**), indicating IR780-ZnS@HSA good stability. Similar to previous research reports (5, 21, 35), the IR780UV absorbance experiment’s loading rate of IR780 was 4.8% (**Figure 3A**). The loading rate of ZnS was 34.23% by inductively coupled plasma mass spectrometry (ICP–MS). As a hydrophobic drug, IR780 has poor stability and can be quickly cleared in the body (36). According to the In vivo delivery requirements, the NPs should have long circulation and pH-responding release abilities (26). **Supplementary Figure 1A** shows the IR780 release curve from IR780-ZnS@HSA in PBS at room temperature. Naked IR780 exhibited an apparent burst release in which most IR780 was released within 2 h. However, only ~15% of IR780 in IR780-ZnS@HSA was released after 72 h, indicating good encapsulation stability of IR780-ZnS@HSA. **Supplementary Figure 1B** is the release curve of the IR780-ZnS@HSA under different pH environments. The release degree gradually increased with decreased pH value. Next, we tested the photoconversion ability of IR780-ZnS@HSA. Compared with the naked IR780, the photothermal properties of IR780-ZnS@HSA did not change significantly (**Figure 3B**). Moreover, photothermal heating/cooling-cycling stability was also demonstrated (**Figure 3C**). The temperature of IR780-ZnS@HSA increased with increased concentration (**Figure 3D**) and power of laser irradiation (**Figure 3E**). Previous studies have shown that IR780 has excellent PDT effects (37, 38). As shown in **Supplementary Figure 2**, the quantitative fluorescence value indicates that IR780-ZnS@HSA with the laser (808 nm) could effectively produce ROS and achieve PDT. The above results indicate that IR780-ZnS@HSA had good stability and photoconversion ability.

### In vitro uptake of IR780-ZnS@HSA

**Figure 4:**
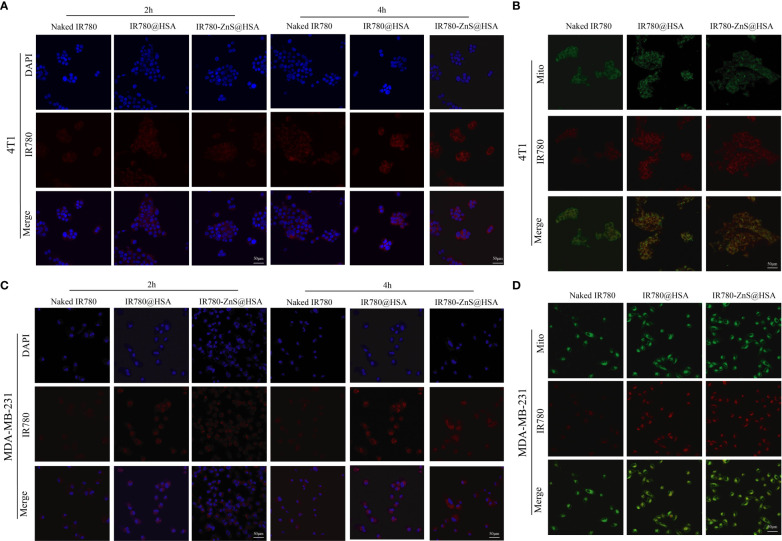
Confocal laser scanning microscopy (CLSM) of the uptake of nanoparticles (NPs) by 4T1 **(A)** and MDA-MB-231 cells **(B)**. CLSM of indicated NPs entering mitochondria after 4 h co-culture with 4T1 **(C)** and MDA-MB-231cells **(D)**. Mito, mitochondria. Scale bar, 50 μm.

CLSM was performed to investigate the uptake ability of IR780-ZnS@HSA by tumor cells. Compared with naked drug IR780, the results showed that MDA-MB-231 and 4T1 cells had more ability for IR780-ZnS@HSA uptake (**Figures 4A, B**, **Supplementary Figures 3A, B** and **Supplementary Figure 4**), which was mainly dependent on the tumor targeting of HSA (24). Studies have shown that HSA can transport substances such as fatty acids, amino acids, and metal ions, showing their potential as drug carriers (39). Numerous studies have confirmed that HSA has the ability to target tumor cells. HSA can passively target tumor cells to avoid phagocytosis by the reticuloendothelial system (40, 41). At the same time, HSA can also bind to related ligands on the tumor cell membrane to achieve active targeting of tumor cells (42). Many studies have shown that IR780 has the ability to mitochondrial targeting (43). CLSM was used to observe the mitochondrial targeting potential of IR780-ZnS@HSA. The results showed that IR780-ZnS@HSA had higher mitochondrial accumulation than naked IR780, while there was no difference between IR780-ZnS@HSA and IR780@HSA (**Figures 3C, D**, **Supplementary Figures 4C, D**). These results suggest that IR780-ZnS@HSA was well engulfed by tumor cells and has a mitochondrial-targeting capacity.

### The antitumor effects of IR780-ZnS@HSA In vitro

**Figure 5:**
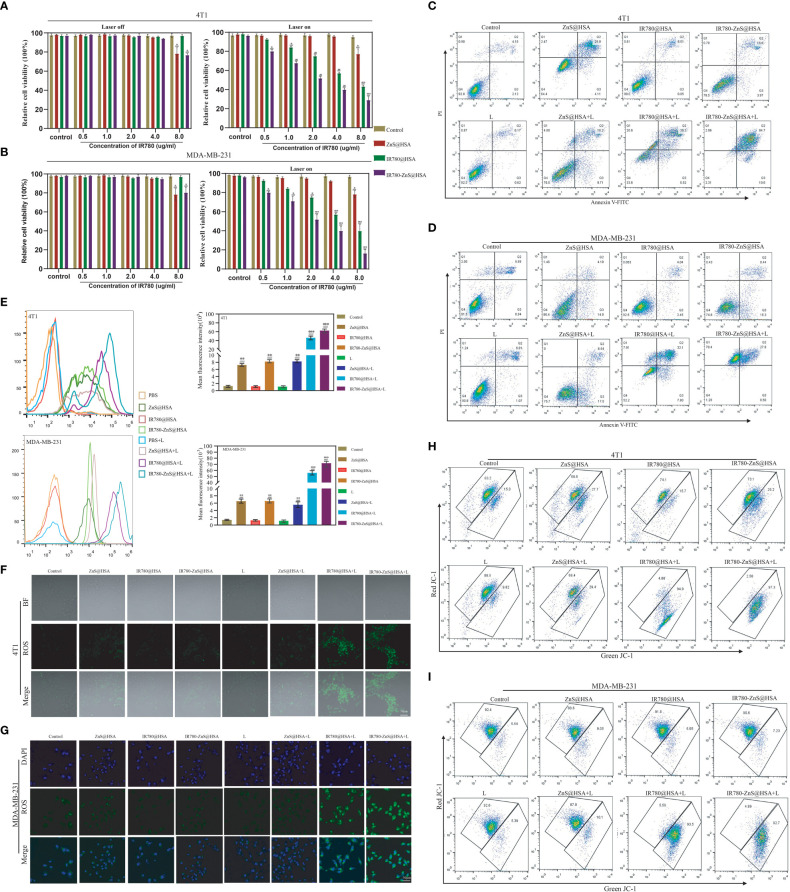
**(A, B)** Viabilities of 4T1 and MDA-MB-231 cells after incubation with different concentrations of various nanoparticles (NPs) with or without 808 nm laser irradiation. **(C, D)** Flow cytometry showing the toxicity in 4T1 and MDA-MB-231 cells co-cultured with different NPs. **(E)** Flow cytometry of reactive oxygen species (ROS) produced in 4T1 and MDA-MB-231 cells co-culture with different NPs. **(F, G)** Confocal laser scanning microscopy (CLSM) of ROS produced in 4T1 and MDA-MB-231 cells. Scale bar, 50 μm. **(H, I)** Flow cytometry of JC-1 in 4T1 and MDA-MB-231 cells co-cultured with different NPs. n = 3, mean ± SD, ANOVA; “###” indicates significant difference compared to the control group. *p < 0.05, **p < 0.01, ***p < 0.001.

The safety of the carrier materials of NDDS is the primary consideration for material selection (44). The survival rate of normal cells (HUVECs) was approximately 100% after incubating HUVECs with IR780-ZnS@HSA at five different concentrations for 24, 48, and 72 h (**Supplementary Figure 5**), indicating excellent biosafety and compatibility of IR780-ZnS@HSA. Next, various methods were used to explore the antitumor effect of IR780-ZnS@HSA In vitro. First, the CCK-8 assay was used to detect the viability of tumor cells treated with different NPs. In the absence of laser irradiation, the toxicity increased with increasing concentrations of IR780-ZnS@HSA in 4T1 and MDA-MB-231 cells (**Figures 5A, B**). However, compared with the control group, the cytotoxicity of IR780-ZnS@HSA was approximately 30% (p < 0.05), which may be due to the high concentration of ZnS required to kill tumor cells effectively (24). However, the toxicity increased significantly with laser irradiation and with increasing concentration of IR780-ZnS@HSA (p < 0.001) (**Figures 5A, B**). Second, flow cytometry was used to detect the cytotoxicity of IR780-ZnS@HSA in tumor cells. Consistent with the results of the CCK-8 assay, compared with the control group, the cytotoxicity of IR780-ZnS@HSA in 4T1 and MDA-MB-231 cells was limited without laser irradiation, and it significantly increased with laser irradiation (**Figures 5C, D** and **Supplementary Figure 6**) IR780-ZnS@HSA to reach more than 90% (p < 0.001). Next, we explored the possible mechanisms of the cytotoxicity of IR780-ZnS@HSA. It has been reported that intracellular ROS, especially those produced by the mitochondria, are involved in regulating cell death (45). Therefore, flow cytometry and CLSM were used to observe ROS changes in tumor cells treated with different NPs. Flow cytometry showed that intracellular ROS in tumor cells treated by IR780-ZnS@HSA + laser was significantly increased compared with the control group (**Figure 5E**) (p < 0.001). The results of CLSM were consistent with those of flow cytometry (**Figures 5F, G** and **Supplementary Figure 7**). It was reported that intracellular zinc ions could produce ROS, which is further facilitated by the generated H_2_S gas from ZnS by inhibiting catalase in tumor cells (24). As shown in **Figure 5E**, intracellular ROS was higher in IR780-ZnS@HSA + laser than in IR780@HSA + laser. Hypoxia is one of the hallmarks of cancer, which restricts PDT effects (5). In this study, IR780-ZnS@HSA was loaded with ZnS, which could produce more ROS, compensating for the limitation of PDT in tumor therapy. It is well known that the decline of mitochondrial membrane potential (MMP) is a hallmark of early apoptosis, and IR780 has mitochondrial targeting properties. Therefore, we assumed that IR780-ZnS@HSA might change MMP and further promote the death of tumor cells. The JC-1 kit was used to detect changes in MMP by flow cytometry. While results showed more green fluorescence in the IR780-ZnS@HSA group, the control group was dominated by red fluorescence, indicating that IR780-ZnS@HSA + laser could decline MMP (**Figures 5G–I** and **Supplementary Figure 8**). Altogether, these results suggest that IR780-ZnS@HSA promotes tumor cell death by targeting mitochondria and declining MMP by increasing intracellular ROS.

### The pyroptosis-inducing potential of IR780-ZnS@HSA

**Figure 6:**
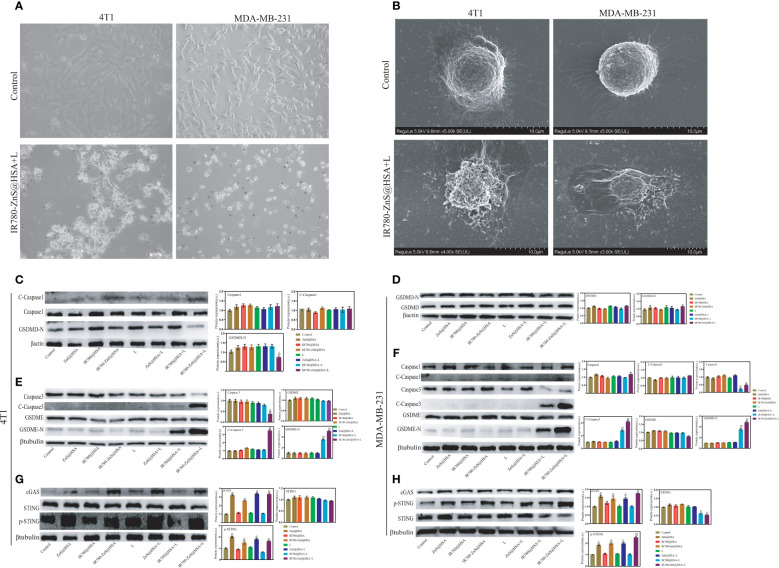
**(A)** Images of 4T1 and MDA-MB-231 cells treated with IR780-ZnS@HSA nanoparticles (NPs). Black arrows indicate dying cells with cell membrane blebbing, ×40. **(B)** Scanning electron microscopy of membrane pores. **(C–H)** The expression level of key proteins in pyroptosis and the cGAS–STING pathway in 4T1 and MDA-MB-231 cells under different treatments. n = 3, mean ± SD, ANOVA; “###” indicates significant difference compared to the control group. **p < 0.01, *p < 0.05, n = 3. L, laser.

Pyroptosis is a new way of cell death that differs from apoptosis. Pyroptosis is inflammatory death and can boost immune response (39). We observed that tumor cells treated with IR780-ZnS@HSA + laser showed balloon-like structures (**Figure 6A**), a typical pyroptosis feature (46, 47), suggesting that IR780-ZnS@HSA may induce pyroptosis. Further, scanning electron microscopy showed numerous pores in the IR780-ZnS@HSA + laser group (**Figure 6B**), compared to a few pores in the control group, which is a typical sign of pyroptosis (48). Studies have shown that PI can penetrate the cell membrane and stain the pyroptotic cell nuclei. Hence, Annexin/PI double staining kit was used to show pyroptosis (13, 49). As shown in **Figures 5C, D**, 4T1 and MDA-MB-231 cells were double stained by Annexin/PI in the IR780-ZnS@HSA + laser group. In order to further verify whether pyroptosis was induced in the IR780-ZnS@HSA + laser group, Western blotting (WB) was used to detect changes in the key proteins of pyroptosis after treatment with different NPs. Previous studies have shown that pyroptosis induction increases the expression of GSDMD, especially GSDMD-N (50). Therefore, we examined changes in the expression of key pyroptosis pathway proteins with different treatments. The results showed no difference in the expression level of GSDMD and GSDMD-N in MDA-MB-231 or 4T1 cells (**Figures 6C–F**). However, recent studies have reported the involvement of other GSDM proteins, such as GSDME, in the regulation of pyroptosis (47). Therefore, we measured the expression level of GSDME and GSDME-N in tumor cells treated with different NPs. Similarly, WB showed no difference in GSDME expression in the different groups (**Figures 6D, E**), while GSDME-N expression increased significantly in the IR780-ZnS@HSA + laser group (**Figures 6E, F**). These results suggest that GSDME might be involved in regulating pyroptosis in tumor cells treated with IR780-ZnS@HSA and laser irradiation. GSDME has been shown to regulate pyroptosis *via* caspase-3 rather than caspase-1 (13, 51). Hence, we measured the expression levels of caspase-3 and cleaved caspase-3 in tumor cells treated with different NPs. Consistent with previous literature reports (13), there was no significant change in caspase-3 (**Figures 6E, F**). However, cleaved caspase-3 (c-caspase-3) significantly increased in the IR780-ZnS@HSA + laser group (**Figures 6E, F**). These results confirmed that IR780-ZnS@HSA + laser induced pyroptosis in tumor cells through the caspase-3–GSDME signaling axis. There are several pathways involved in pyroptosis, including the caspase-1-GSDMD classical pathway, caspase4/5/11-mediated non-classical pyroptosis, and caspase-3-dependent pyroptosis signaling pathway (52). Unlike GSDMD-dependent pyroptosis induced by caspase-1 or caspase-4/5/11, caspase-3 promotes the recruitment of the GSDME-N domain to the cell membrane by cutting off GSDME and induces the formation of cell pores, thus leading to pyroptosis (13). Studies have shown an effective antitumor strategy by activating the caspase-3–GSDME pathway (53). Meanwhile, we measured the expression levels of cGAS, STING, and p-STING proteins in tumor cells treated with different NPs by WB based on previous studies showing cGAS–STING signaling axis activation by Zn^2+^ (24). We showed that the expression level of STING did not increase in tumor cells treated with different NPs, while cGAS and p-STING increased significantly (**Figures 6G, H**). Previous studies have shown that when the cGAS–STING pathway was activated, the level of IFN-β increased and then mediated innate and acquired immunity (54). As shown in **Supplementary Figure 9**, NPs that could activate the cGAS–STING pathway significantly increased the level of extracellular IFN-β. Much evidence shows that the body’s immune system is regulated by Zn^2+^ (55). Lack of Zn^2+^ leads to immune dysfunction (56). Studies have shown that Zn^2+^ can activate innate immune response through and cGAS–STING pathway. The specific mechanism is that Zn^2+^ promotes cGAS phase transition in the presence of cytosolic DNA and then increases the enzymatic activity of cGAS (57). Cen et al. showed that ZnS@BSA nanoparticles activated the cGAS–STING pathway through Zn^2+^ to enhance immune response, showing an excellent antitumor effect (24). In summary, we confirmed that IR780-ZnS@HSA + laser could induce pyroptosis through the caspase-3–GSDME signaling axis and activate the cGAS–STING signaling axis in tumor cells.

### IR780-ZnS@HSA induces ICD in tumor cells

**Figure 7:**
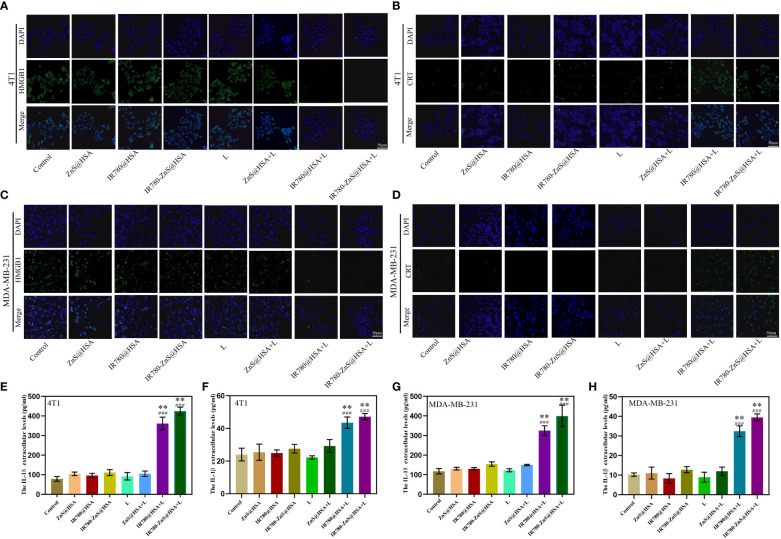
Confocal laser scanning microscopy (CLSM) of high-mobility group box 1 (HMGB1) in 4T1 **(A)** and MDA-MB-231 cells **(B)** co-cultured with different nanoparticles (NPs). Scale bar, 50 μm. CLSM of calreticulin (CRT) in 4T1 cells **(C)** and MDA-MB-231 cells **(D)** co-cultured with different NPs. Scale bar, 50 μm. Cell supernatant levels of IL-18 **(E, G)** and IL-1β **(F, H)** in 4T1 and MDA-MB-231 cells under different treatments. n = 3, mean ± SD, ANOVA; “###” indicates significant different from control group. *p < 0.05, **p < 0.01.

We confirmed that IR780-ZnS@HSA could induce pyroptosis in tumor cells in the abovementioned assays. However, whether pyroptosis was an ICD in tumor cells was unclear. A series of studies have shown that HMGB1 and CRT are typical markers of ICD. ICD is always accompanied by the exposure of CRT on the cell surface and the extracellular release of HMGB1 (27). CLSM was used to observe the changes in HMGB1 and CRT in tumor cells treated with different NPs. Consistent with previous reports (27), the expression of HMGB1 was decreased in the MDA-MB-231 and 4T1 cells of the IR780-ZnS@HSA + laser group (**Figures 7A, B**, **Supplementary Figures 10A, B**), while the CRT was increased mainly on the cell membrane (**Figures 7C, D**, **Supplementary Figures 10C, D**). IL-18 and IL-1β are classical inflammatory markers (58, 59) and were measured in the supernatant of tumor cells treated with different NPs using ELISA assay. The results showed that IL-18 in the IR780-ZnS@HSA + laser group was almost twofold higher than that in the control group in 4T1 and MDA-MB-231 cells (**Figures 7E, G**) (p < 0.01). Similarly, the expression level of IL-1β was higher in the IR780-ZnS@HSA + laser group than in the control group (**Figures 7F, H**) (p < 0.01). These results suggest that IR780-ZnS@HSA with laser irradiation can induce ICD in tumor cells.

### Transcriptome sequencing (RNA-seq)

RNA-seq refers to high-throughput sequencing of mRNA and non-coding RNA (ncRNA) in cells. Compared with the whole genome and whole exome, RNA-seq contains more gene expression and sequence information, which is beneficial for analyzing gene fusion, splicing variation, and gene expression profile (60). To further investigate the antitumor mechanism of IR780-ZnS@HSA, RNA-seq was performed in 4T1 cells treated by IR780-ZnS@HSA + laser (experimental group), with PBS as the control group. The differences between the treated tumor cells and the control group were analyzed using the Kyoto Encyclopedia of Genes and Genomes (KEGG).

**Figure 8:**
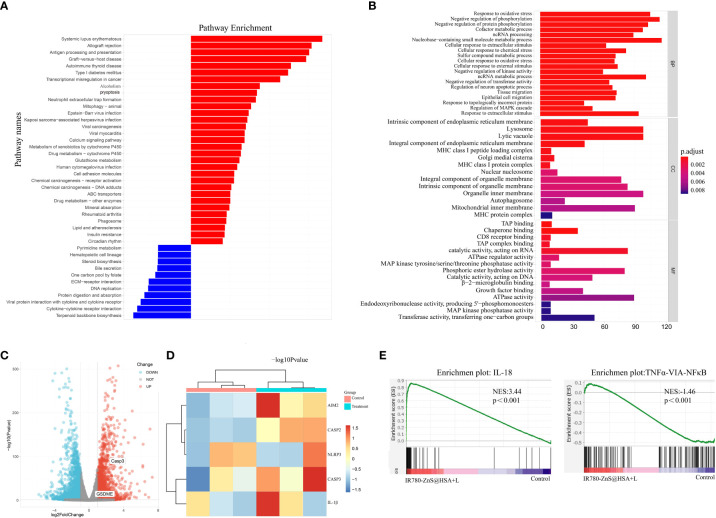
**(A)** Kyoto Encyclopedia of Genes and Genomes (KEGG) enrichment analysis from IR780-ZnS@HSA and control treatments. **(B)** KEGG enrichment analysis from IR780-ZnS@HSA and control treatments. **(C)** Volcano plots of IR780-ZnS@HSA and control treatments. **(D)** Heat maps of differentially expressed pyroptosis genes in 4T1 cells treated with IR780-ZnS@HSA or placebo. **(E)** Gene Set Enrichment Analysis (GSEA) from IR780-ZnS@HSA and control treatments.

Compared with the control group, a total of 3,567 transcripts were altered in the experimental group, including 1,791 upregulated and 1,776 downregulated transcripts. KEGG and Gene Ontology (GO) enrichment analysis showed that the pathways of pyroptosis, inflammation, and antigen presentation were enriched in the IR780-ZnS@HSA + laser group (**Figures 8A, B**). These data confirm the inflammatory effect and cytotoxicity of IR780-ZnS@HSA in tumor cells, which can be attributed to the pyroptotic effects of IR780-ZnS@HSA in tumor cells. The volcano plot and heat map of differentially expressed genes associated with programmed cell death is shown in **Figures 8C, D**, respectively. The noticeable expression changes and Gene Set Enrichment Analysis (GSEA) (**Figure 8E**) confirmed that IR780-ZnS@HSA could induce pyroptosis and inflammatory responses in tumor cells.

### In situ antitumor activity of IR780-ZnS@HSA

**Figure 9:**
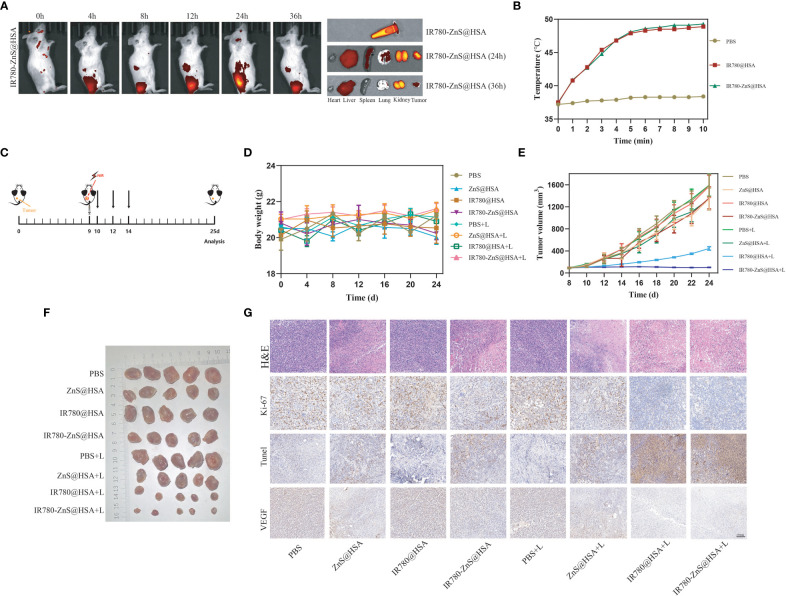
**(A)** Distribution of IR780-ZnS@HSA in mice at different time points (2, 4, 8, 12, 24, and 36 h) after tail vein injection. **(B)** The temperature changes of mice after different drugs were injected through the tail vein with 808 nm laser irradiation. **(C)** Schematic diagram of tumor inoculation and administration into 4T1 tumor-bearing mice for *in situ* analysis. **(D)** Body weight changes of mice in different treatment groups. **(E)** Tumor images of the different treatment groups of mice. **(F)** Changes in tumor volume in eight groups during treatment. **(G)** Hematoxylin and eosin (H&E) and immunohistochemical staining (Ki-67, TdT-mediated dUTP-nick end labeling (TUNEL), and vascular endothelial growth factor (VEGF)) of mice treated with different drugs (scale bar, 100 μm).

We have explored the mechanism by which IR780-ZnS@HSA exerts its antitumor effects In vitro. Next, we wanted to investigate the antitumor effect In vivo of IR780-ZnS@HSA. To test the tumor-targeting specificity IR780-ZnS@HSA In vivo, IR780-ZnS@HSA or PBS was injected into the 4T1 tumor model through the tail vein. The specificity of IR780-ZnS@HSA at the tumor site was detected by fluorescence imaging after 2, 4, 8, 12, 24, and 36 h. The results showed that IR780-ZnS@HSA accumulated at the tumor site in a time-dependent manner, reaching a peak at 24 h and then gradually decreasing (**Figure 9A**). However, due to the non-tumor specificity of naked IR780, no accumulation of IR780 was observed at the tumor site after 36 h (**Supplementary Figure 11**). The tumor targeting of IR780-ZnS@HSA significantly improved after using HSA as the nanocarrier, providing a basis for the antitumor effect In vivo.

We then used the photothermal imaging system to observe temperature changes in the tumor site of tumor-bearing mice with laser irradiation 24 h after injecting IR780-ZnS@HSA or PBS. The results showed that the temperature of the tumor site could reach 45°C with 5-min laser irradiation, while the temperature of the control group slightly increased (**Figure 9B**).

In order to explore the antitumor effect of IR780-ZnS@HSA, 4T1 tumor cells were injected subcutaneously into the left dorsal side of balb/c mice to establish mice 4T1 syngeneic model. When the tumor volume reached 70–100 mm^3^, the mice were randomly divided into eight groups (n = 7 per group) and treated with different NPs (**Figure 9C**). There was no significant difference in body weight among the groups (**Figure 9D**). At the predetermined observation endpoint, the tumor growth in the PBS group, PBS + laser group, and IR780@HSA group increased rapidly. In contrast, noticeable growth inhibition of the primary tumor was achieved in the IR780-ZnS@HSA + laser group (**Figures 9E, F**). H&E staining was performed in tumor tissue sections. There was more nuclear pyknosis, karyorrhexis, and karyolysis in the IR780-ZnS@HSA + laser group (**Figure 9G**). Ki-67 staining, a nuclear protein used to evaluate the proliferation capacity of cancers (61), was applied to tumor tissue sections of the different groups. The level of Ki-67 was significantly decreased in the IR780-ZnS@HSA + laser group compared with the control group (**Figure 9G**). TUNEL assay is a method for detecting the toxicity of tumor cells (62), where dark brown nuclei indicate cell death. As shown in **Figure 9G**, more cell death was detected in the IR780-ZnS@HSA + laser group, as indicated by the significant number of dark brown nuclei detected in the IR780-ZnS@HSA + laser group, compared to little, if any, in the control group IR780-ZnS@HSA. A series of studies have shown that vascular endothelial growth factor (VEGF) plays a crucial role in tumor neovascularization, invasion, and metastasis (63). Immunohistochemistry was used to observe the expression of VEGF in different groups. As shown in **Figure 9G**, VEGF significantly decreased in the IR780-ZnS@HSA + laser group, suggesting that IR780-ZnS@HSA + laser can inhibit tumor angiogenesis. Taken together, the above results suggest that IR780-ZnS@HSA-mediated PTT/PDT can effectively suppress tumor growth.

### IR780-ZnS@HSA triggers the immune response In vivo

In the abovementioned assays, we confirmed that IR780-ZnS@HSA could induce pyroptosis, thus leading to robust ICD In situ. Many studies have shown that ICDs are closely related to immunotherapy efficacy in cancers (64, 65). Based on our findings, we explored the impact of IR780-ZnS@HSA + laser on improving aPD-L1 efficacy.

**Figure 10:**
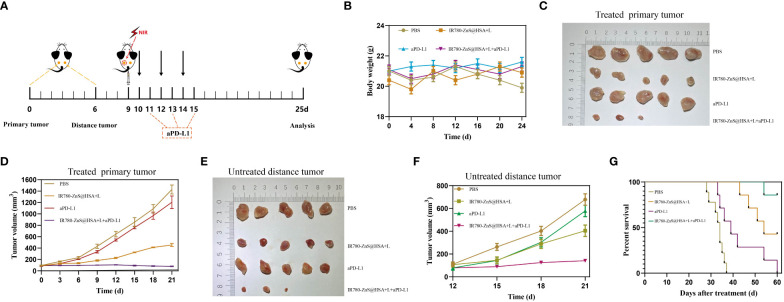
**(A)** Schematic diagram of tumor inoculation and administration into 4T1 tumor-bearing mice for abscopal effect. **(B)** Body weight changes of 4T1 tumor-bearing mice in different treatment groups. Typical photographs of primary **(C)** and distant tumors **(E)** collected on day 25. Tumor growth curves of primary **(D)** and distant tumors **(F)** in 4T1 tumor-bearing mice after various treatments. **(G)** Survival of mice was continuously monitored for 60 days, and mice were automatically considered dead once tumor volume exceeded 2,000 mm^3^ (n = 8 per group).

The bilateral 4T1 tumor model was first constructed. When the primary tumor (left dorsal) volume reached 70–100 mm^3^, the mice were randomly divided into four groups (n = 7 per group) and administered with different treatments (**Figure 10A**). During the treatment, there was no significant difference in the body weight of mice between the groups (**Figure 10B**). At the predetermined observation endpoint, the primary tumor growth in the control group and aPD-L1 group increased rapidly. In contrast, noticeable growth inhibition of the primary tumor was observed in the IR780-ZnS@HSA + laser group and was more apparent in the IR780-ZnS@HSA + laser + aPD-L1 group, especially in the IR780-ZnS@HSA + laser + aPD-L1 group, where tumors were in complete remission in two of the mice (**Figures 10C, D**). The growth of the untreated distant tumors was monitored after administering different treatments to the primary tumors. The results showed that the distant tumors were efficiently suppressed in the IR780-ZnS@HSA + laser group and IR780-ZnS@HSA + laser + aPD-L1 group, especially in the IR780-ZnS@HSA + laser + aPD-L1 group, where the tumors were in complete remission in two of the mice (**Figures 10E, F**). Further, the survival time of the mice was assessed after the various treatments. Mice in the control groups had an average life span of 28 to 40 days. In contrast, mice in the IR780-ZnS@HSA + laser + aPD-L1 group survived over 60 days (**Figure 10G**).

**Figure 11:**
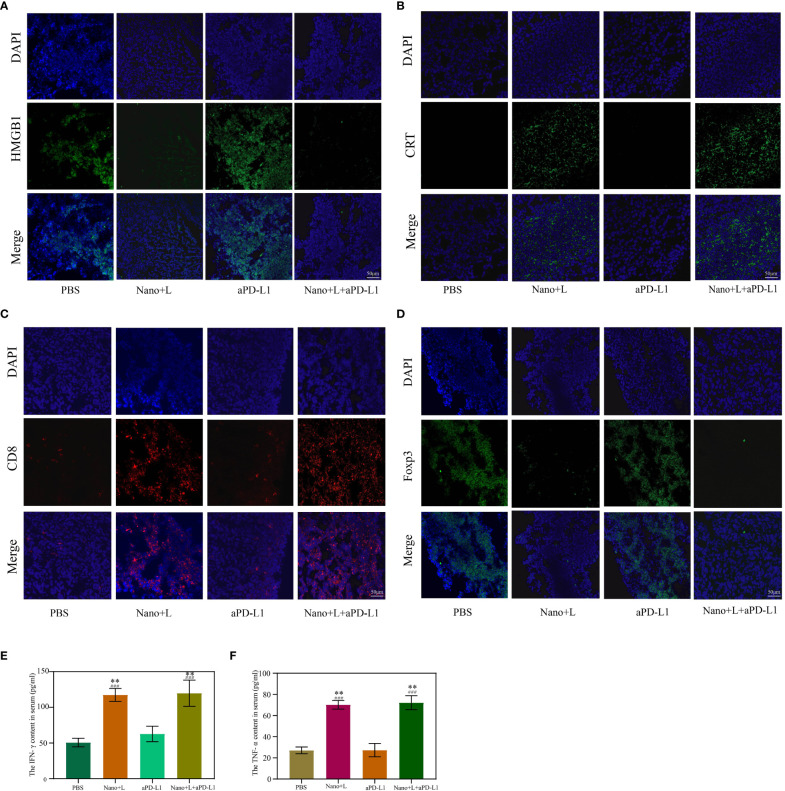
Immunofluorescence staining of high-mobility group box 1 (HMGB1) **(A)** and calreticulin (CRT) **(B)** in the primary tumor on day 7. Scale bar, 50 μm. Immunofluorescence staining of Foxp3 **(C)** and CD8 **(D)** in the primary tumor on day 7. Scale bar, 50 μm. **(E, F)** Serum cytokine levels of IFN-γ and TNF-α at day 3 after various treatments; “###” indicates significant difference compared to the control group. n = 3, mean ± SD, ANOVA, **p < 0.01. Nano, IR780-ZnS@HSA; L, laser.

Next, the mechanism of IR780-ZnS@HSA-mediated photo-immunotherapy was evaluated In vivo. First, CLSM was used to observe ICD markers in tumor tissue sections of different groups. The results showed that HMGB1 decreased in IR780-ZnS@HSA + laser and IR780-ZnS@HSA + laser + aPD-L1 group (**Figure 11A** and **Supplementary Figure 12A**), while the CRT increased (**Figure 11B** and **Supplementary Figure 12B**), indicating a robust induction of ICD in tumor tissues in the IR780-ZnS@HSA + laser and IR780-ZnS@HSA + laser + aPD-L1 groups. Numerous studies have shown that immune cell infiltration in tumor tissues is critical in immunotherapy (66, 67). Tumor-associated immune cells can exhibit both tumor-antagonizing and tumor-promoting functions, depending on the characteristics of the TME (68). Helper T cells (CD3^+^ CD4^+^) contribute to the process of immune regulation and can be divided based on their function into two subtypes, effector T cells (CD3^+^ CD4^+^ FoxP3^−^) and regulatory T cells (Tregs) (CD3^+^ CD4^+^ Foxp3^+^). Effector T cells boost while Tregs suppress the antitumor immune response (27).

We explored whether the IR780-ZnS@HSA + laser treatment could shape the landscape of the infiltrating immune cells in the TME by simultaneously measuring the immunofluorescent intensities of three parameters (CD4, CD8, and Foxp3) in the tissue samples. The mice were subjected to PBS or aPD-L1 treatment. A large number of Foxp3+ cells infiltrated the tumor sites (**Figure 11D** and **Supplementary Figure 12D**), suggesting the existence of a strong immunosuppressive TME. In contrast, the IR780-ZnS@HSA + laser and IR780-ZnS@HSA + laser + aPD-L1 groups markedly increased the CD8^+^ T cells while decreasing the FoxP3^+^ T cells in the tumors (**Figure 11C** and **Supplementary Figure 12C**).

Finally, the serum levels of several immune-related cytokines (27), including IFN-γ and tumor necrosis factor (TNF-α), were measured by ELISA. Mice in the IR780-ZnS@HSA + laser and IR780-ZnS@HSA + laser + aPD-L1 groups exhibited higher levels of serum IFN-γ and TNF-α compared with those in the other two groups (**Figures 11F,G**) (p < 0.01), indicating a robust immunomodulatory effect.

Altogether, these findings suggest that IR780-ZnS@HSA-based treatment might remodel the TME by increasing CD8^+^ T-cell infiltration and decreasing immunosuppressive Tregs, thereby changing TNBC from a “cold” tumor to a “hot” tumor (69–71).

### Biosafety assay of IR780-ZnS@HSA

A favorable biosafety profile is an essential prerequisite for any approved clinical drug (27), particularly for immunotherapeutic drugs, where excessive amplification of the immune response may cause severe side reactions. We evaluated the biosafety of IR780-ZnS@HSA after showing its excellent tumor inhibition In vivo IR780-ZnS@HSA. The hemolysis assay showed a hemolysis rate of IR780-ZnS@HSA less than 5% in different concentration groups (**Supplementary Figures 13A, B**), which is lower than the standard threshold of 5% (72). Therefore, there was no hemolysis in the IR780-ZnS@HSA group, indicating an excellent hemocompatibility of IR780-ZnS@HSA. In addition, the body weight recorded every 3 days showed no significant difference in the different groups (**Figures 9D**, **10B**). Moreover, H&E staining was performed on the major organs (heart, liver, spleen, lung, and kidney), indicating no abnormalities in different groups (**Supplementary Figure 13C**). Finally, no difference in complete blood and biochemistry analysis was observed in the different groups at the end of treatment (**Supplementary Figures 13D–I**). These results indicated a favorable biocompatibility of IR780-ZnS@HSA.

TNBC is a highly malignant tumor with a poor prognosis. Therapeutic methods for this tumor include surgery, chemoradiotherapy, targeted therapy, and immunotherapy (73). However, single antitumor therapy has certain limitations in overcoming recurrence and metastasis. The combination of existing therapies to improve the efficacy of TNBC is a current focus of TNBC research (74). Nevertheless, combination therapy may increase toxic side effects, and some may even be fatal (75). In this study, we innovatively loaded both immunomodulators and photothermal conversion agents into the nanocapsules and simultaneously measured local PTT/PDT and systemic immunotherapy. Local phototherapy (PT) therapy and dual remission of immunosuppression (i.e., PD-1/PD-L1 checkpoint blocking and activating cGAS–STING) were highly effective treatment strategies for both primary and metastatic tumors. Studies have shown that immune⁃related adverse events (IRAEs) cannot be ignored during immunotherapy. The overall IRAE incidence was approximately 66%, while its incidence in grades 3 and above was approximately 14.0%, indicating partially fatal events (75, 76). The IRAE was higher in combination therapies. However, in this study, IR780-ZnS@HSA + laser combined with aPD-L1 showed no apparent toxic side effects. Our results indicated that IR780-ZnS@HSA could be effective in antitumor therapy with good biological safety. Moreover, these results provide new directions for the treatment of TNBC.

## Conclusion

In this study, we designed and developed IR780-ZnS@HSA, a new inducer of pyroptosis with the trinity of PTT/PDT/immunotherapy by self-assembly approach. First, the non-toxic HSA was used as a nanocarrier, ensuring the biosafety of IR780-ZnS@HSA. Moreover, the tumor-targeting ability of HSA promotes the intracellular uptake of IR780-ZnS@HSA. Second, IR780 with laser irradiation can be accumulated in the mitochondria of tumor cells, inducing mitochondrial dysfunction and cytotoxicity. In addition, intracellular zinc ions can produce ROS, further facilitated by the generated H_2_S gas from ZnS, which inhibits catalase under acidic TME in tumor cells. The excessive ROS can compensate for the attenuated PDT effect caused by hypoxia in the tumor. Third, IR780-ZnS@HSA can induce pyroptosis in tumor cells through the caspase-3–GSDME pathway, thus leading to robust ICD In situ. Furthermore, IR780-ZnS@HSA can activate the cGAS–STING pathway by upregulating innate immunity. Consequently, the activated caspase-3–GSDME and cGAS–STING pathways boost the immune response, significantly inhibiting the primary and distant metastatic tumors. As a new multifunctional pyroptosis inducer, IR780-ZnS@HSA is a simple and inexpensive NP that warrants further investigations in tumor-targeted immunotherapy.

## Data availability statement

The datasets presented in this study can be found in online repositories. The names of the repository/repositories and accession number(s) can be found below: https://www.ncbi.nlm.nih.gov/bioproject/, PRJNA971787.

## Ethics statement

The animal study was reviewed and approved by Sichuan Cancer Hospital.

## Author contributions

JY and ML planned and designed the experiments and drafted the main part of the manuscript. ML provided funding to perform this study. JY, WG, RH, and JB performed the In vitro study and analyzed the data. JY, WG, RH, TW, SZ, and CH performed the In vivo study and analyzed the data. ZH, JL, and CZ analyzed the data. All authors read and approved the final manuscript.
