# Supplementary material for: Self-assembled albumin nanoparticles induce pyroptosis for photodynamic/photothermal/immuno synergistic therapies in triple-negative breast cancer
Source: Front Immunol. 2023 May 26;14:1173487. doi: 10.3389/fimmu.2023.1173487 (PMC10279487; doi:10.3389/fimmu.2023.1173487)
Supplement: Supplementary file 1 [file DataSheet_1.docx]

***Supplementary Material***


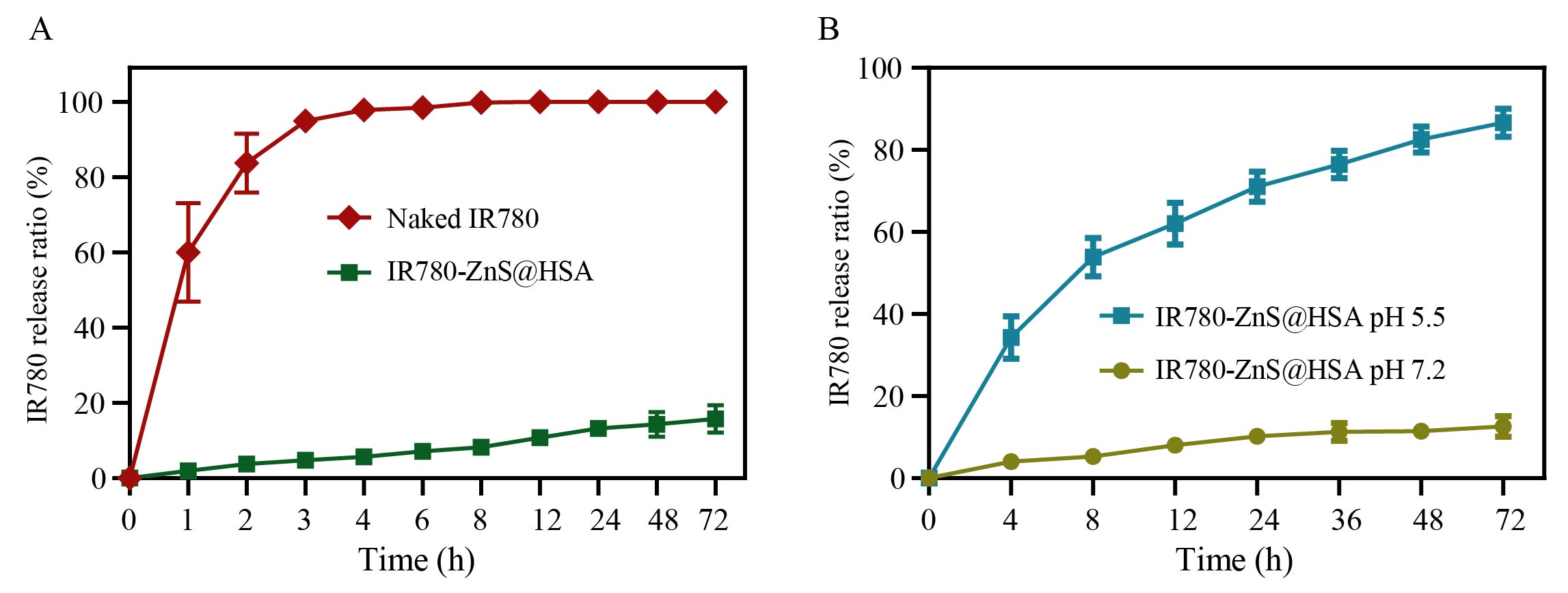
**SUPPLEMENTARY FIGURE 1. (A)** In vitro IR780 release profile of Naked and IR780-ZnS@HSA. **(B)** In vitro pH-dependent IR780 release profile of IR780-ZnS@HSA.


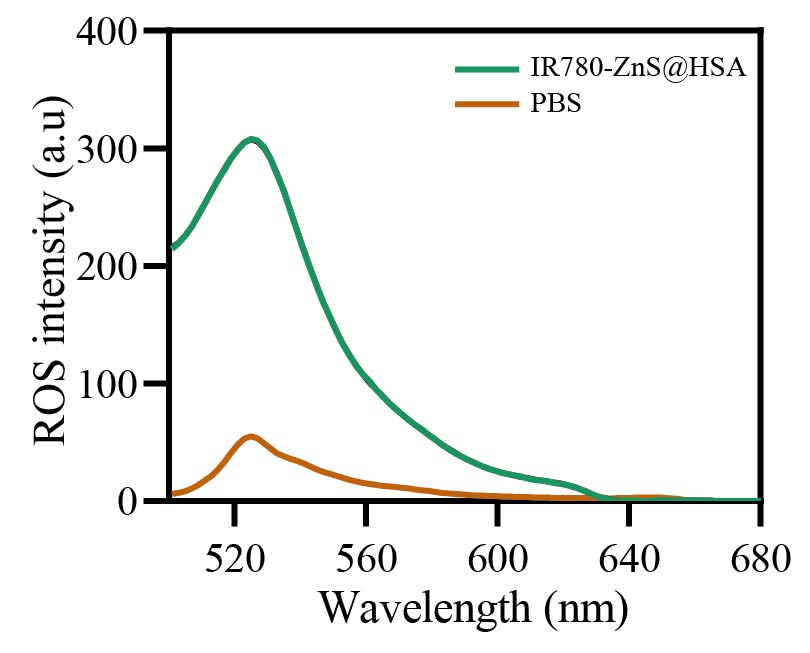


**SUPPLEMENTARY FIGURE 2.** Fluorescence emission of ROS at 525 nm after incubated with IR780-ZnS or PBS under 808nm laser (1W cm^2^) irradiation for 5 min.


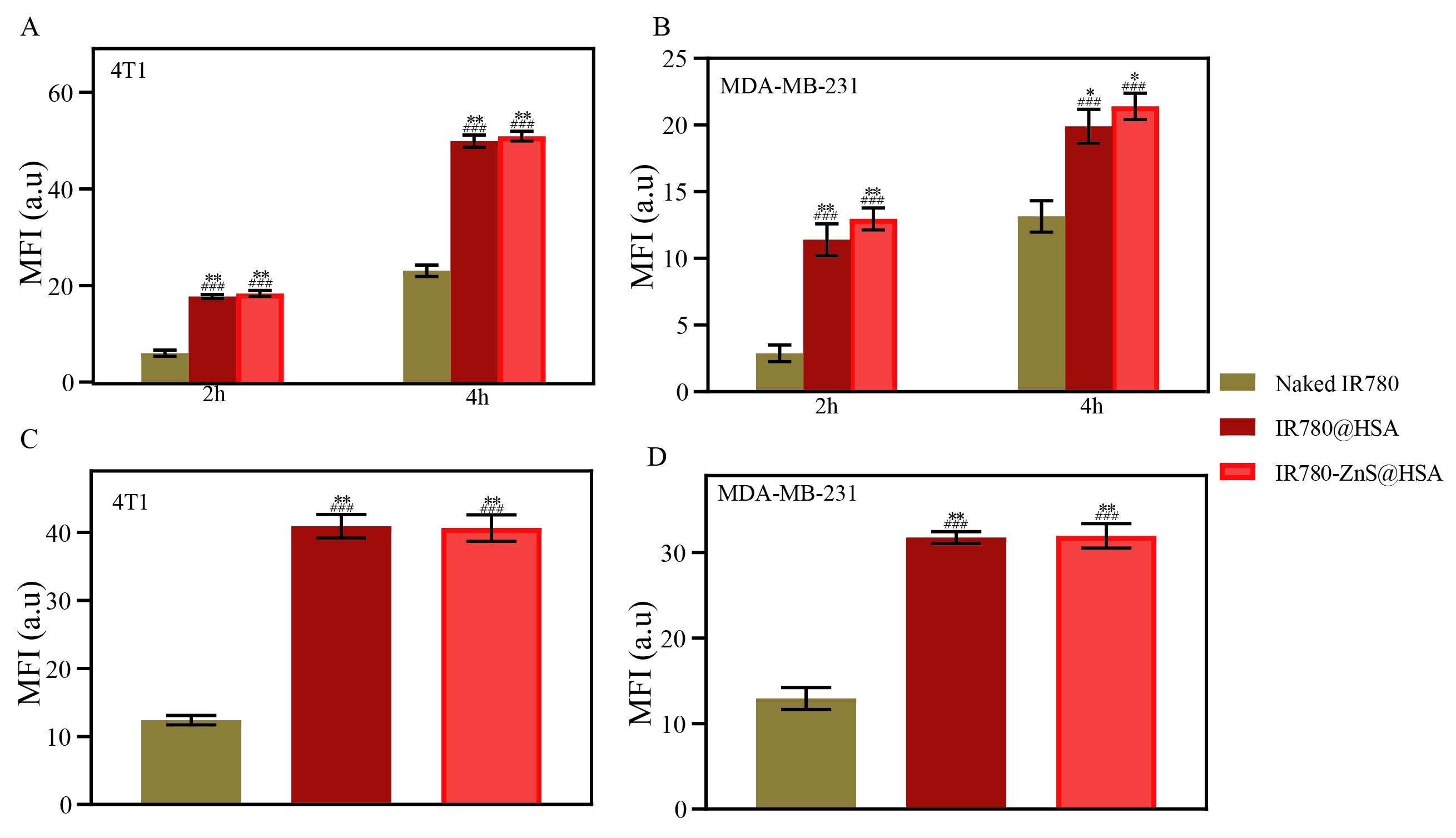


**SUPPLEMENTARY FIGURE 3.** **(A)** Quantification of intracellular IR780 in 4T1 cells for 2 and 4 h. **(B)** Quantification of intracellular IR780 in MDA-MB-231 cells for 2 and 4 h. **(C)** Quantification of intramitochondrial IR780 in 4T1 cells for 4 h. (**D**) Quantification of intramitochondrial IR780 in MDA-MB-231 cells for 4 h. n=3, mean ± SD, ANOVA, “**###**” significant different from control group, *****P<0.05, ******P<0.01.


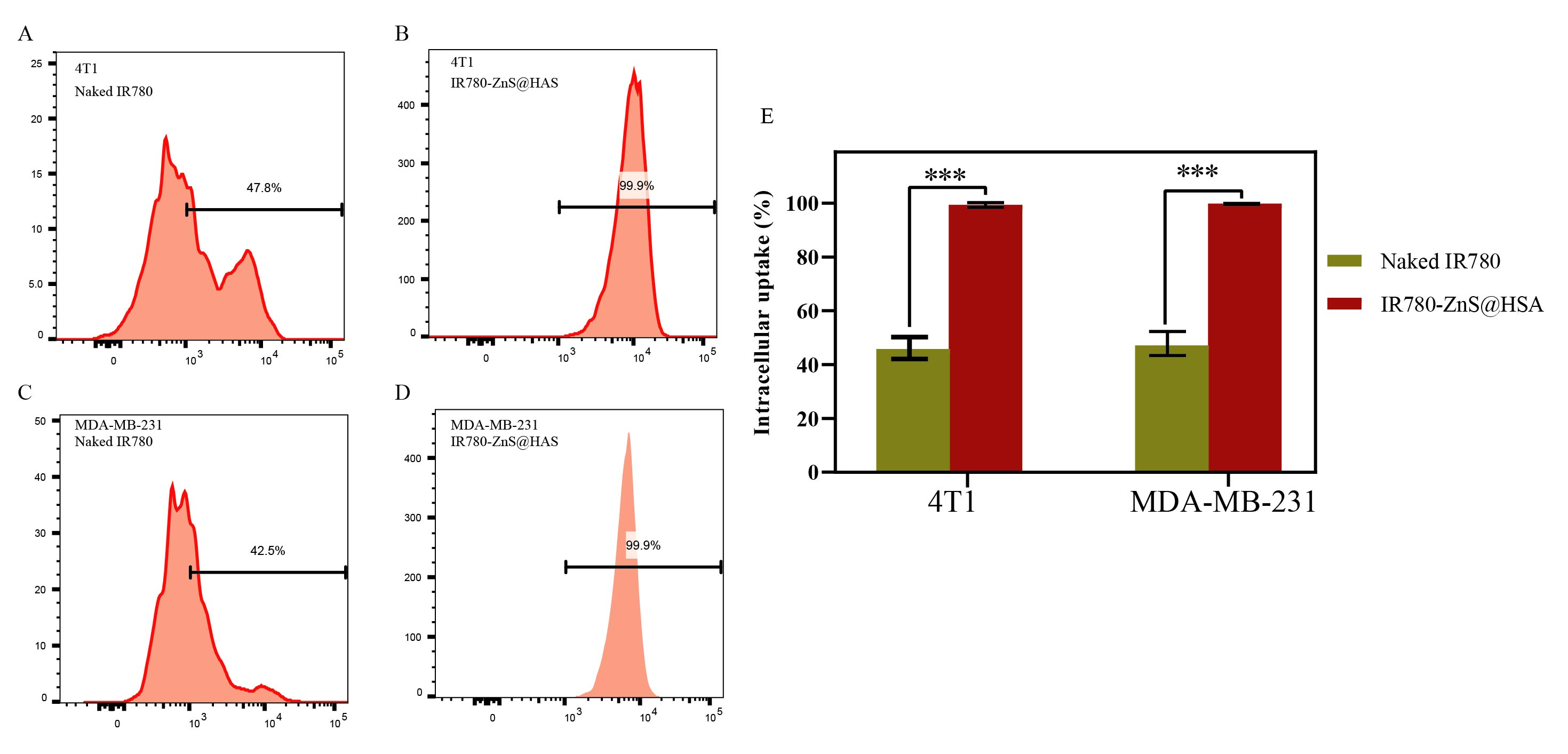


**SUPPLEMENTARY FIGURE 4. A and C** Flow cytometry analysis of intracellular uptake of Naked IR780 after 4 h co-culture with 4T1 and MDA-MB-231. B **and D** Flow cytometry analysis of intracellular uptake of IR780-ZnS@HSA after 4 h co-culture with 4T1 and MDA-MB-231. **E** Statistical analysis of Figure (a-d), n=3, mean ± SD, ANOVA, *******P<0.001.


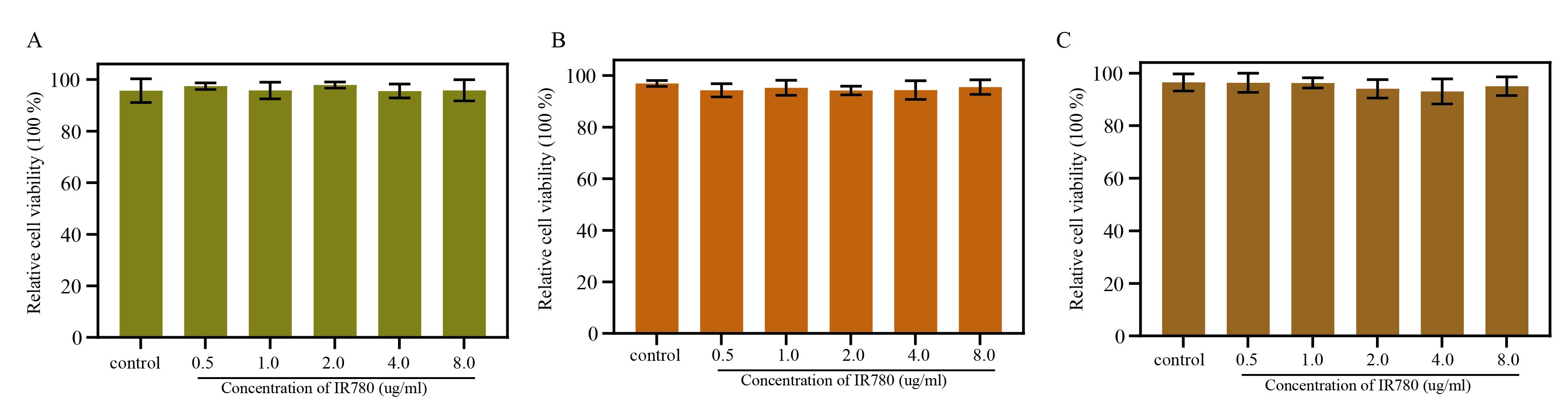


**SUPPLEMENTARY FIGURE 5.** Safety evaluation of IR780-ZnS@HSA on HUVECs incubated for 24h **(A)**, 48h **(B)**, and 72h **(C)** ( n=3, mean ± SD, ANOVA).


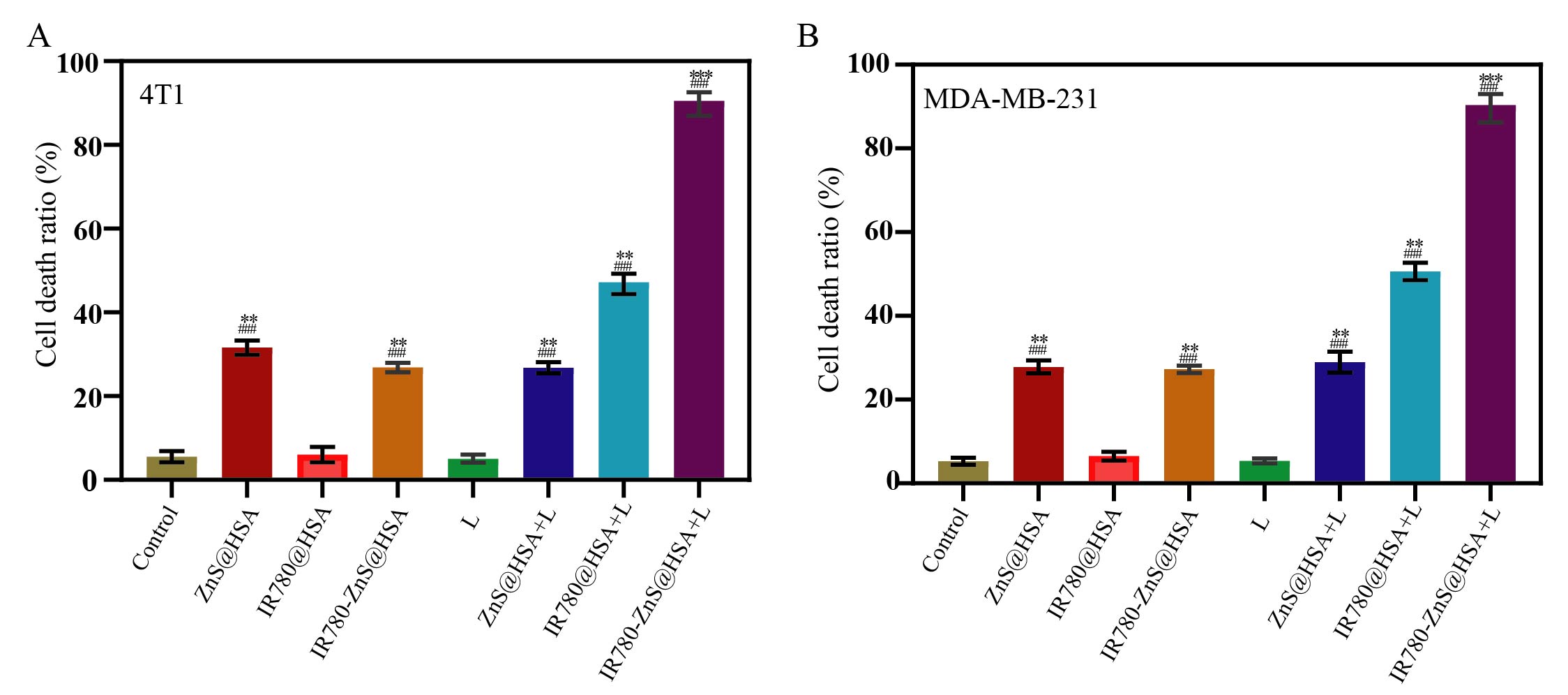


**SUPPLEMENTARY FIGURE 6.** **(A)** Percentage of cell death in figure 5C. **(B)** Percentage of cell death in figure 5D. n=3, mean ± SD, ANOVA, “**###**” significant different from control group, ******P<0.01, *******P<0.001. L, Laser.


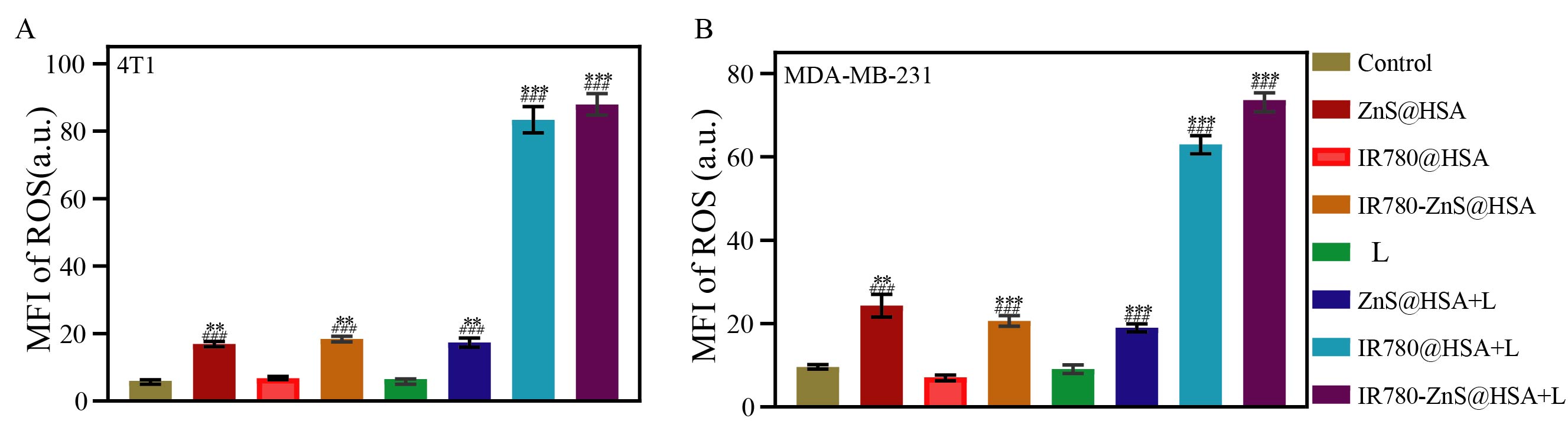


**SUPPLEMENTARY FIGURE 7.** **(A)** Quantification of ROS in figure 5F. **(B)** Quantification of ROS in figure 5G. n=3, mean ± SD, ANOVA, “**###**” significant different from control group, ******P<0.01, *******P<0.001. L: Laser.


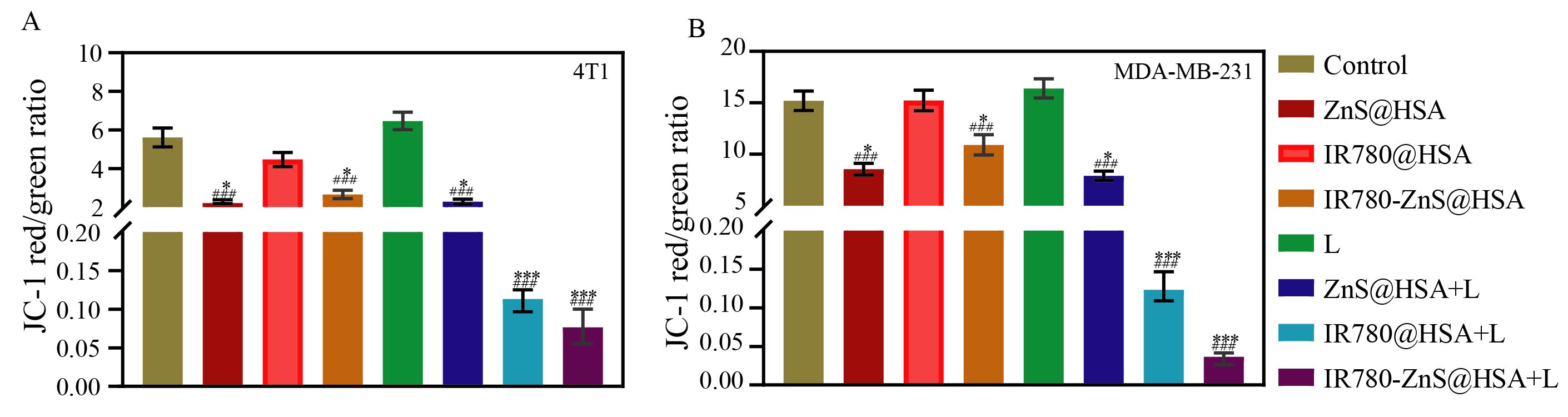


**SUPPLEMENTARY FIGURE 8.** Percentage of JC-1 red/green in 4T1**(A)** and MDA-MB-231**(B)**, n=3, mean ± SD, ANOVA, “**###**” significant different from control group, ******P<0.01, *******P<0.001. L: Laser.


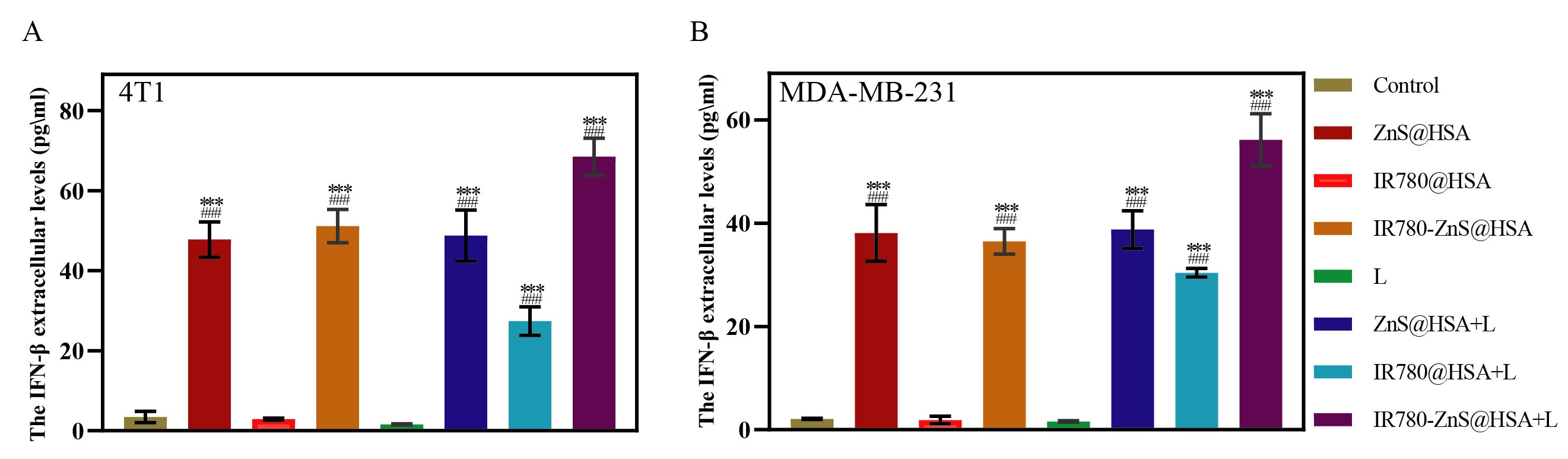


**SUPPLEMENTARY FIGURE 9.** Cell supernatant levels of IFN-β in 4T1(A) and MDA-MB-231 cells(B) under different treatments. n=3, mean ± SD, ANOVA, “###” significant different from control group. ***P<0.001. L, Laser.


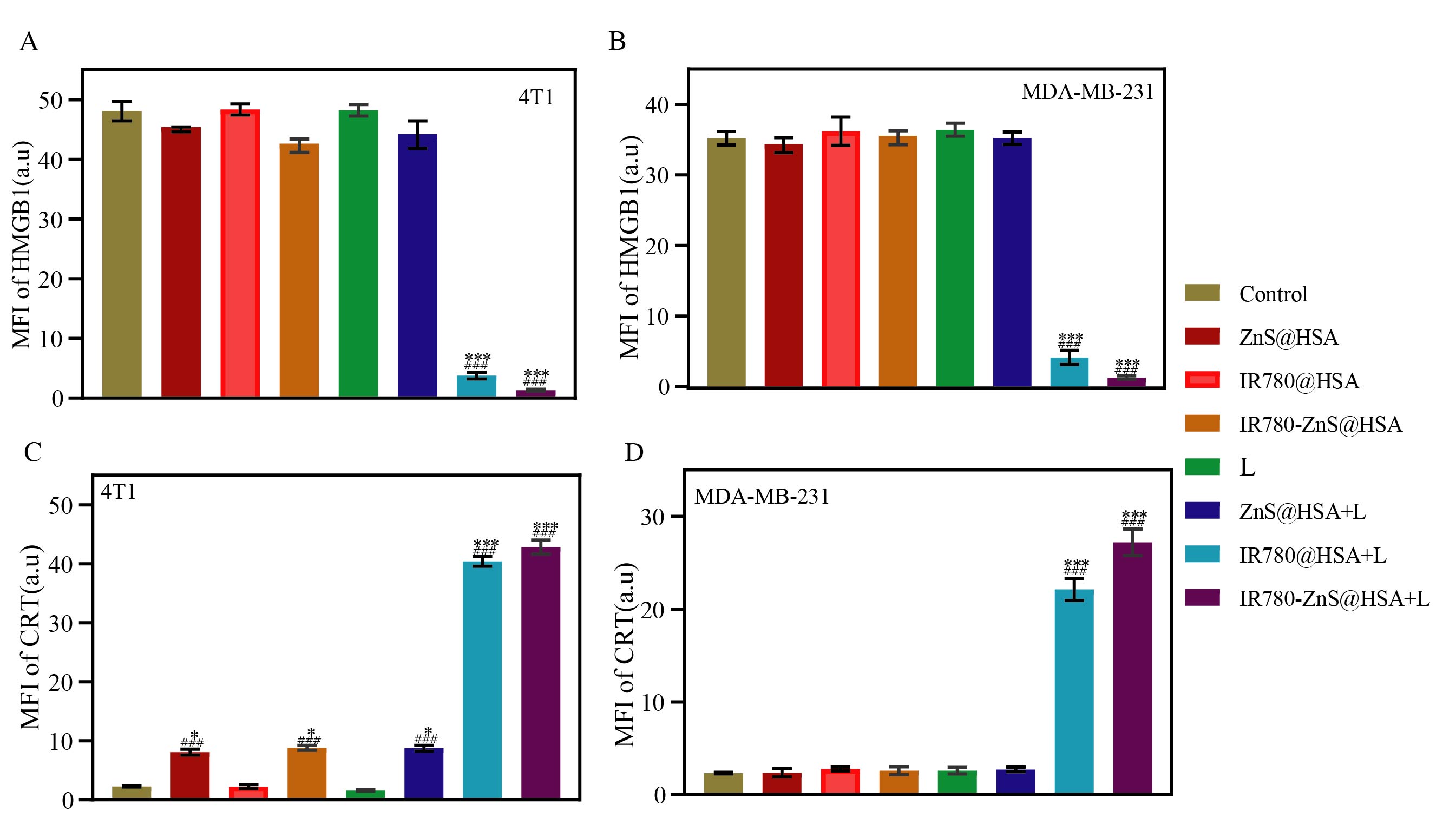


**SUPPLEMENTARY FIGURE 10**. Mean fluorescence intensity (MFI) of HMGB1 in 4T1**(A)** and MDA-MB-231 cells **(B)** was quantified from n=3 independent experiments. MFI of CRT in 4T1**(C)** and MDA-MB-231 cells **(D)** was quantified from n=3 independent experiments.n=3, mean ± SD, ANOVA, “**###**” significant different from control group, *****P<0.05, *******P<0.001. L: Laser.


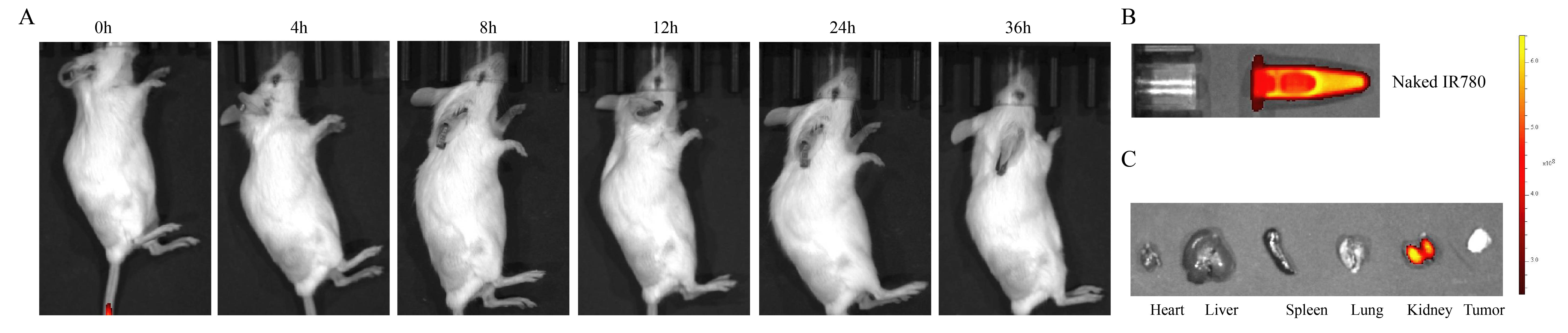


**SUPPLEMENTARY FIGURE 11.** (A) In vivo FL imaging after naked IR780 injection at different time points (0, 2, 4, 8, 12, 24 and 36 h). **(B)** The FL imaging of naked IR780. **(C)** Ex vivo FL images of tumor tissue and major organs of mice after 24 h post injection of IR780-ZnS@HSA.


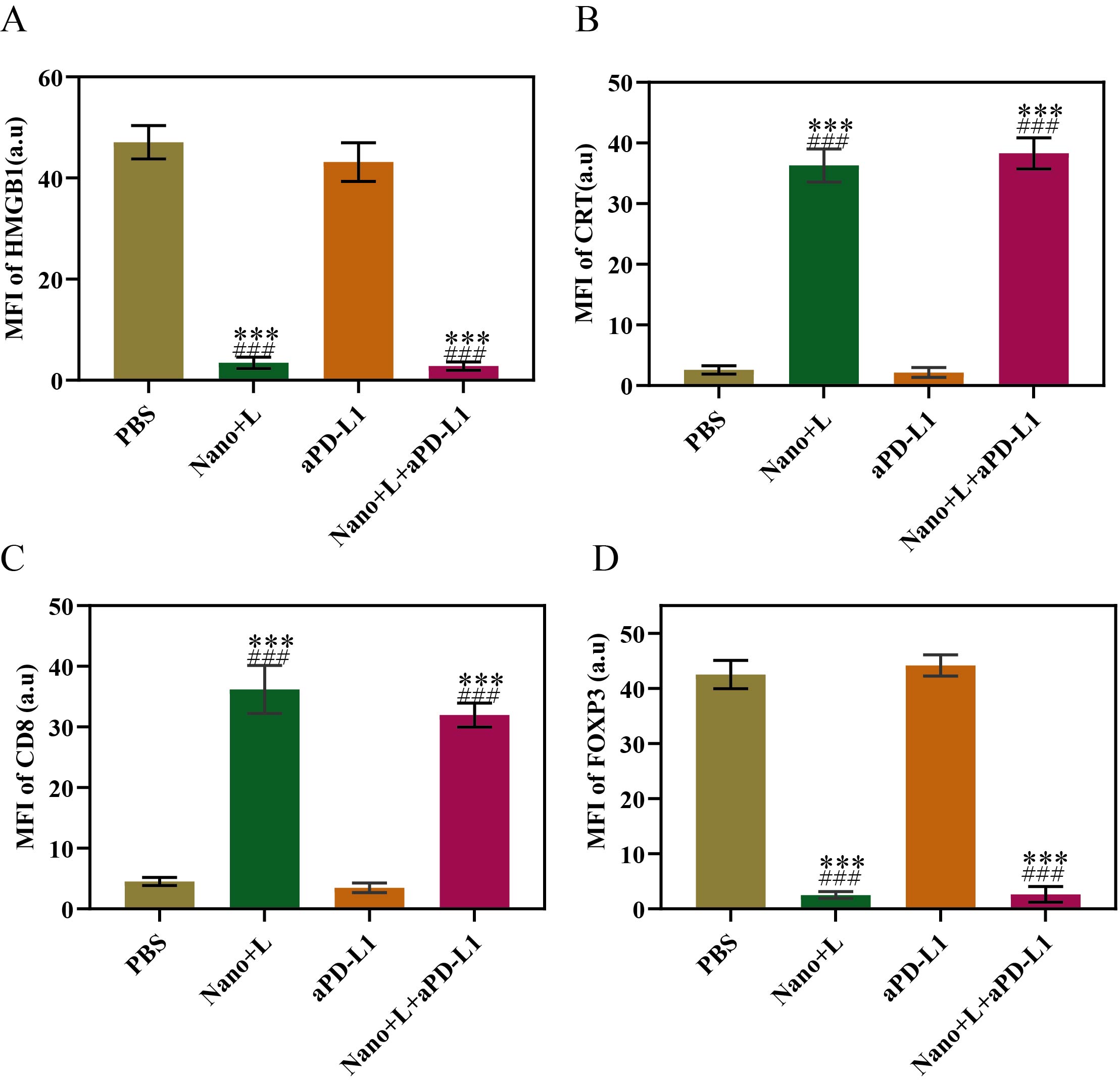


**SUPPLEMENTARY FIGURE 12.** (A) Mean fluorescence intensity (MFI) of HMGB1. (B) MFI of CRT. (C) MFI of CD8. (D) MFI of FOXP3. n=3, mean ± SD, ANOVA, “**###**” significant different from control group, *******P<0.001. Nano, IR780-ZnS@HSA. L, Laser.


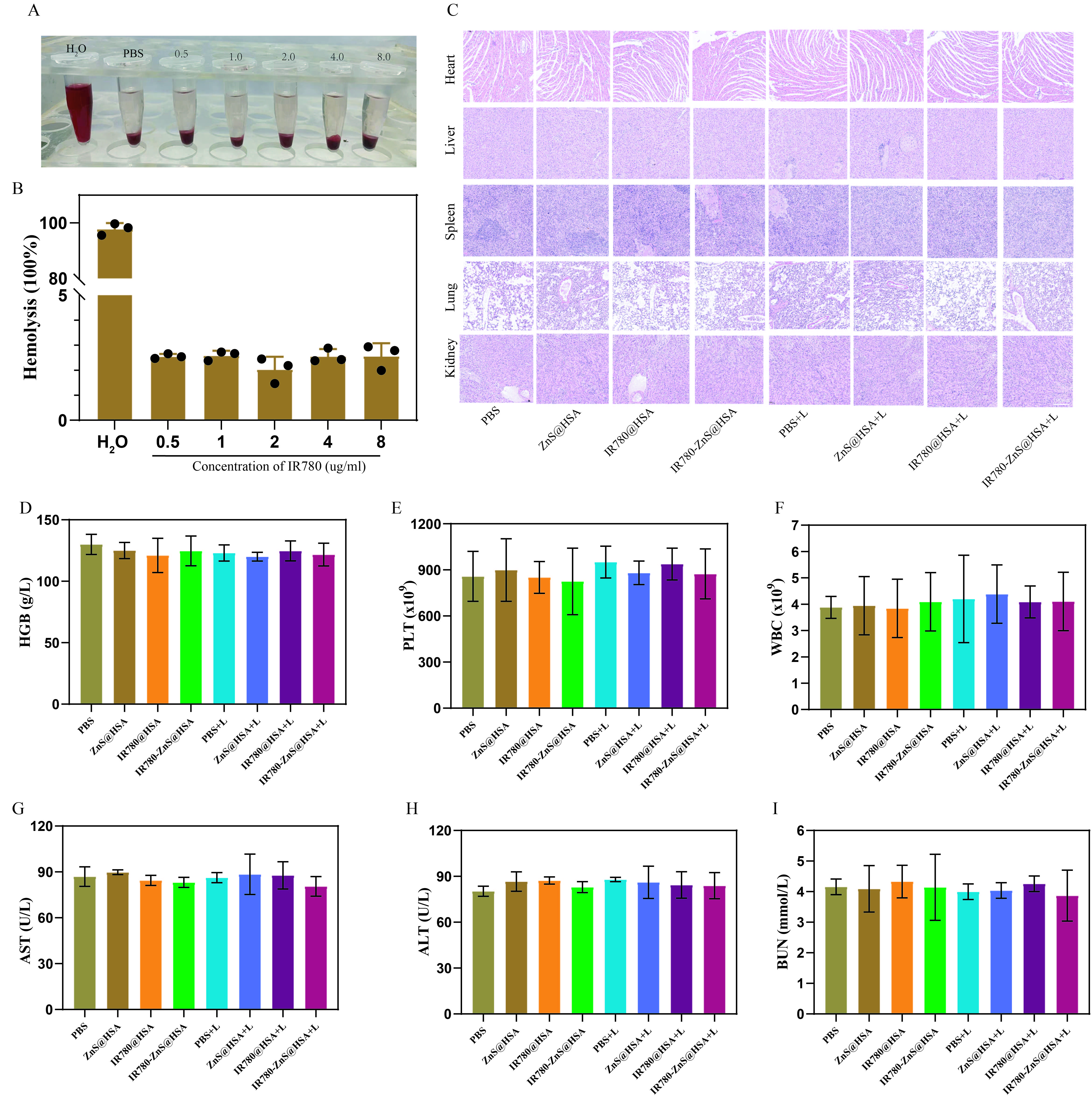


**SUPPLEMENTARY FIGURE 13.** (A) Representative photographs of hemolysis of RBCs incubated with various concentrations of IR780-ZnS@HSA for 3 h. DIW and PBS dispersed RBCs were served as positive and negative control, respectively. (B) Hemolysis ratios of RBCs after incubation with various concentrations of IR780-ZnS@HSA for 3 h (n=3). (C) HE staining (lung, liver, spleen, kidney, heart) of mice treated with indicated drugs (scale bar: 100μm). (D-I) Complete blood analysis and blood biochemistry of 4T1 tumor-bearing mice after various treatment. L: Laser.
